# CGRP signalling inhibits NO production through pannexin-1 channel activation in endothelial cells

**DOI:** 10.1038/s41598-019-44333-w

**Published:** 2019-05-28

**Authors:** Pablo S. Gaete, Mauricio A. Lillo, Mariela Puebla, Inés Poblete, Xavier F. Figueroa

**Affiliations:** Departamento de Fisiología, Facultad de Ciencias Biológicas, Pontificia Universidad Católica de Chile, Santiago, 8330025 Chile

**Keywords:** Extracellular signalling molecules, Hypertension, Peripheral vascular disease, Vasodilation

## Abstract

Blood flow distribution relies on precise coordinated control of vasomotor tone of resistance arteries by complex signalling interactions between perivascular nerves and endothelial cells. Sympathetic nerves are vasoconstrictors, whereas endothelium-dependent NO production provides a vasodilator component. In addition, resistance vessels are also innervated by sensory nerves, which are activated during inflammation and cause vasodilation by the release of calcitonin gene-related peptide (CGRP). Inflammation leads to superoxide anion (O_2_^• −^) formation and endothelial dysfunction, but the involvement of CGRP in this process has not been evaluated. Here we show a novel mechanistic relation between perivascular sensory nerve-derived CGRP and the development of endothelial dysfunction. CGRP receptor stimulation leads to pannexin-1-formed channel opening and the subsequent O_2_^• −^-dependent connexin-based hemichannel activation in endothelial cells. The prolonged opening of these channels results in a progressive inhibition of NO production. These findings provide new therapeutic targets for the treatment of the inflammation-initiated endothelial dysfunction.

## Introduction

Vascular endothelium plays a key role in the control of vasomotor tone through the production of vasoactive substances^1,2^. In resistance arteries and arterioles, the main endothelium-derived vasodilators are nitric oxide (NO) and a signal known as endothelium-derived hyperpolarizing factor (EDHF)^2^, which depends on the activation of Ca^2+^-activated K^+^ channels of small and intermediate conductance in endothelial cells^3,4^. Although endothelial cells may also produce prostacyclin, this prostaglandin does not contribute to the endothelium-dependent vasodilation in normal conditions^5^. In addition, peripheral blood vessels are densely innervated by sympathetic and sensory nerves. In contrast to sympathetic nerves, sensory C and Aδ fibres express transient receptor potential V1 (TRPV1) channels, and then, can be activated with capsaicin, a selective agonist of these channels^6^. In resistance arteries, the main neurotransmitter released by capsaicin-sensitive sensory endings is the calcitonin gene-related peptide (CGRP)^7^, which is a potent vasodilator. Consequently, it is thought that sensory nerves are involved in the control of vasomotor tone^8–10^.

Interestingly, pro-inflammatory stimuli activate the release of CGRP from sensory nerves^11,12^. Inflammation is associated with the development of an endothelial dysfunction characterized by a reduction in the NO-mediated vasodilation induced by endothelium-dependent vasodilators such as acetylcholine (ACh)^13^, which is thought to be mediated by an increase in reactive oxygen species^14,15^. Furthermore, we recently showed that transient activation of perivascular sensory nerves with capsaicin leads to a gradual reduction in the expression of endothelial nitric oxide synthase (eNOS)^16^, which suggests that activation of sensory nerves may mediate the endothelial dysfunction observed in inflammation.

The development of inflammation has been associated in several cell types with opening of connexin (Cx)-based hemichannels or pannexin (Panx)-formed channels^17–19^. Connexins are typically recognized as the protein subunit of intercellular gap junction channels. However, gap junctions are composed of two hemichannels, which can also function independently and allow the exchange of ions and small molecules (<1.4 nm of diameter) between the intra and extracellular compartments^20,21^. Five connexin isoforms have been identified in blood vessels: Cx32, Cx37, Cx40, Cx43 and Cx45, but only the expression of Cx37, Cx40, Cx43 and Cx45 has been confirmed in resistance arteries^22–28^. On the other hand, pannexins are a protein family structurally related to connexins that form membrane channels with similar characteristic of hemichannels^29^. Of the three pannexin isoforms described (Panx-1, Panx-2 and Panx-3), only Panx-1 is consistently found in endothelial and smooth muscle cells of resistance vessels of the peripheral circulation^30^. Deregulated activity of connexin hemichannels or Panx-1 channels has been linked to cell dysfunction or cell death^31–33^. Interestingly, CGRP induces an increase of Panx-1 channel opening in mesenteric resistance arteries^16^; however, the functional significance of this process remains to be determined.

In this work, we evaluated the participation of CGRP release from capsaicin-sensitive perivascular sensory nerves in the control of NO signalling in mesenteric resistance arteries. We found that transient stimulation with capsaicin initiates a CGRP-mediated progressive inhibition of NO production and the subsequent NO-dependent vasodilation through a signalling mechanism triggered in endothelial cells by the activation of Panx-1 channels, which, in turn, leads to a superoxide anion (O_2_^• −^)-dependent opening of connexin-formed hemichannels.

## Results

**Figure 1:**
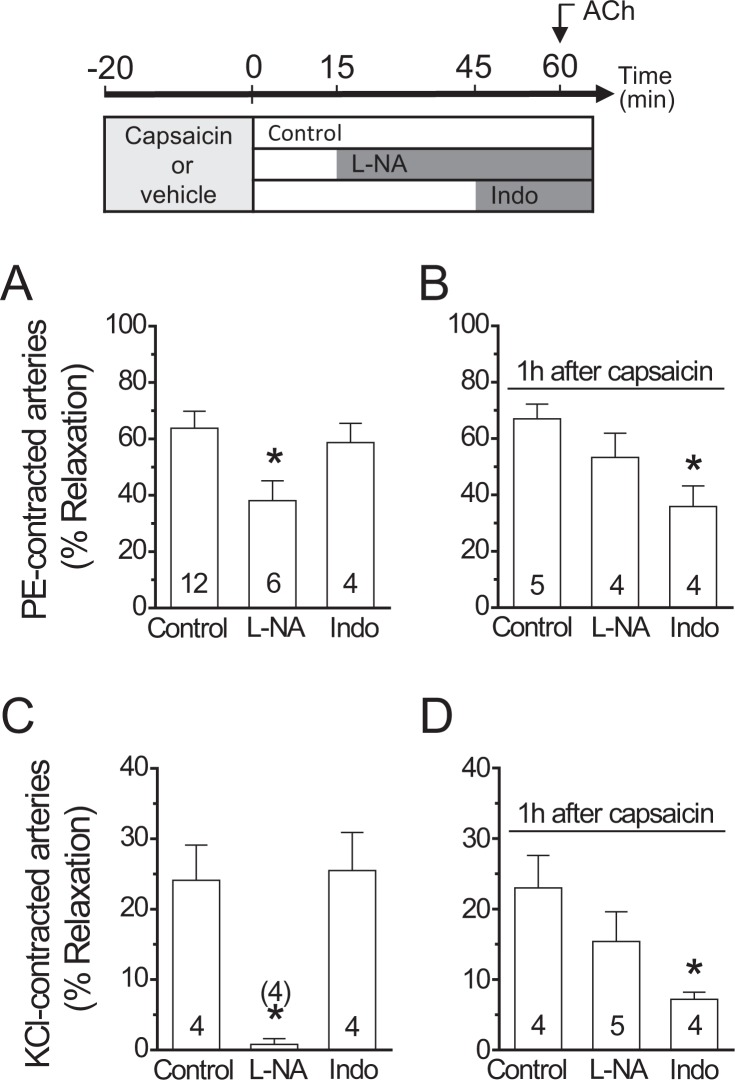
Activation of capsaicin-sensitive sensory nerves triggers the inhibition of ACh-induced NO-dependent vasodilation. The experimental protocol of these results is depicted on the top of the figure. (**A**) Relaxation induced by 100 nM ACh in phenylephrine (PE)-contracted mesenteric arteries in control conditions and after blockade of NO production with 100 µM N^G^-nitro-L-arginine (L-NA) or prostaglandin formation with 10 µM indomethacin (Indo). (**B**) NO- and prostaglandin-dependent vasodilator components activated by ACh 1 h after capsaicin treatment (1 µM, 20 min) in PE-contracted arteries. (**C**) ACh-elicited relaxation of KCl-contracted mesenteric arteries in the absence and presence of L-NA or Indo. (**D**) L-NA and Indo sensitive vasodilator components evoked by ACh 1 h after capsaicin treatment in KCl-contracted arteries. Note that the capsaicin-triggered NO signalling inhibition was fully compensated by the activation of an indomethacin-sensitive vasodilator component. Numbers inside the bars or in parentheses indicate the n value. Values are means ± SEM. *P < 0.05 vs control by one-way ANOVA plus Dunnett post hoc test.

Basal perfusion pressure of the isolated mesenteric arterial beds was 4.6 ± 0.8 mmHg and, in control conditions, application of 60 µM phenylephrine (PE) elicited an increase in perfusion pressure of 20.1 ± 3.6 mm Hg to stabilize in 25.2 ± 3.9 mm Hg after 3 to 4 min. Stimulation with 100 nM ACh for 1 min induced a rapid reduction in perfusion pressure (Figs 1A and S1), as a consequence of the relaxation of resistance arteries of the mesenteric arterial bed. As expected, the ACh-induced relaxation was attenuated by the blockade of NO production with 100 µM N^G^-nitro-L-arginine (L-NA), but not by the inhibition of prostaglandin production with indomethacin (Figs 1A and S1).

### NO signalling inhibition by capsaicin-sensitive sensory nerves

**Figure 2:**
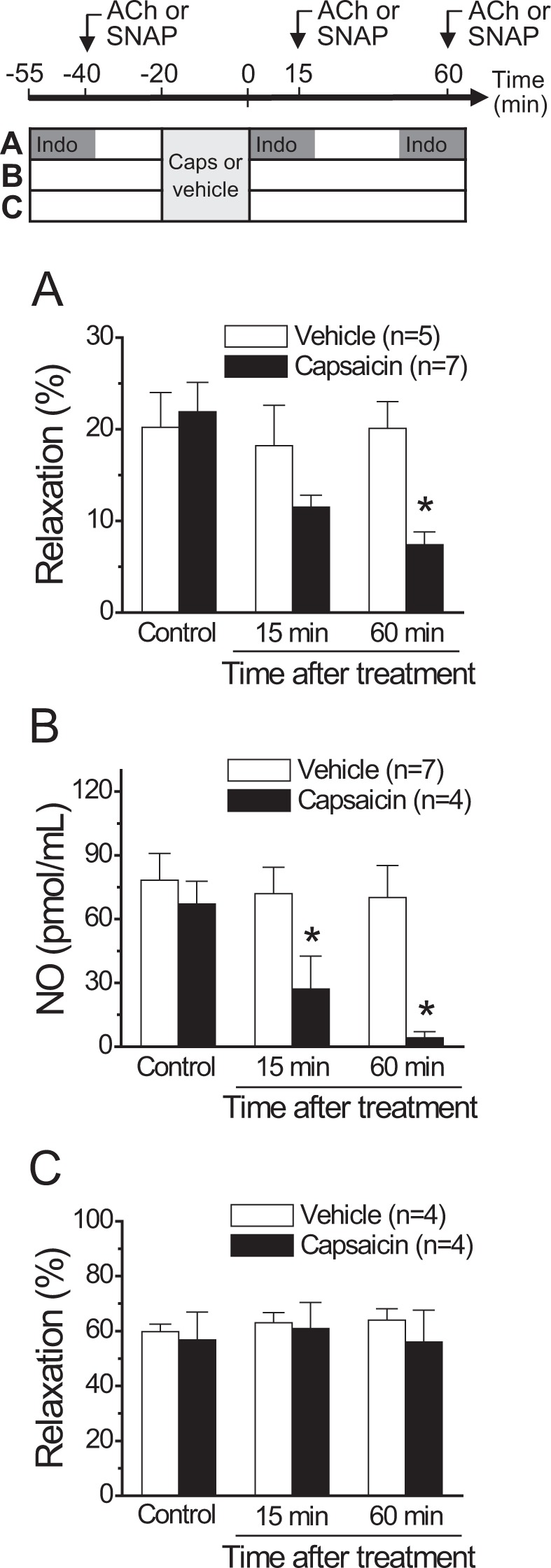
Time course of the capsaicin-initiated inhibition of the ACh-induced NO-dependent vasodilator component. The experimental protocol of the results shown in A, B and C is depicted on the top of the figure. (**A**) Relaxation induced by 100 nM ACh in KCl-contracted mesenteric arteries in control conditions and 15 or 60 min after treatment with capsaicin (1 µM) or its vehicle for 20 min. ACh was applied in the presence of 10 µM indomethacin to prevent the interference of prostaglandins in the response. (**B**) NO production induced by successive ACh (100 nM) stimulations along the time in mesenteric arterial beds treated with capsaicin or the vehicle. (**C**) Relaxation induced by 300 nM SNAP, a NO donor, in KCl-contracted mesenteric arteries before (control) or 15 or 60 min after treatment with capsaicin or its vehicle. Values are means ± SEM. *P < 0.05 vs control by one-way ANOVA plus Dunnett post hoc test.

The NO-dependent vasodilator component observed in control conditions declined after the activation of sensory nerves by 20 min application of 1 µM capsaicin and, consequently, 1 h after this treatment, the ACh-induced relaxation was resistant to L-NA, but, in addition, it was sensitive to indomethacin (Figs 1B and S2A). Interestingly, the magnitude of the inhibition attained in the presence of L-NA in control conditions was similar to the indomethacin-sensitive component observed after capsaicin treatment (Fig. 1A,B). As capsaicin did not affect the maximal response induced by ACh (Figs 1B and S2B), these results indicate that capsaicin-mediated sensory nerve activation did not alter the EDHF-dependent vasodilator component of the response. Therefore, to analyse the effect of capsaicin on the NO-dependent vasodilation without the interference of the EDHF signalling, we contracted the mesenteric arteries with 70 mM KCl (Fig. 1C,D), which evoked an increase in perfusion pressure of 22.0 ± 5.9 mmHg over baseline. Consistent with the inactivation of the EDHF-mediated relaxation, the response to 100 nM ACh in these vessels was smaller than that observed in PE-contracted mesenteries and the vasodilation depended exclusively on NO, as demonstrated by the complete blockade of the response with L-NA in control conditions (Fig. 1C). As observed in PE-contracted preparations, the NO-dependent vasodilator component was replaced by an indomethacin-sensitive vasodilation 1 h after the treatment with capsaicin (Fig. 1D). The analysis of the vasomotor responses in the presence of indomethacin revealed that the capsaicin-triggered NO signalling inhibition was developed slowly along the time, as depicted by the progressive decay of the ACh-induced vasodilation and NO production observed 15 and 60 min after the treatment with capsaicin (Fig. 2A,B). However, the prostaglandin-dependent compensatory vasodilator pathway was not observed 15 min after capsaicin application (Supplementary Fig. S2C), indicating that this vasodilator component did not arise in parallel to the inhibition of the NO signalling, but with a delay. In contrast to the response induced by ACh, transient capsaicin application did not affect the vasodilator response evoked by 300 nM S-nitroso acetyl penicillamine (SNAP), a NO donor (Fig. 2C). As expected, the vasodilation elicited by SNAP was similar in KCl- and PE-contracted mesenteries (Supplementary Fig. S3).

### Capsaicin-induced changes in eNOS phosphorylation via CGRP release

**Figure 3:**
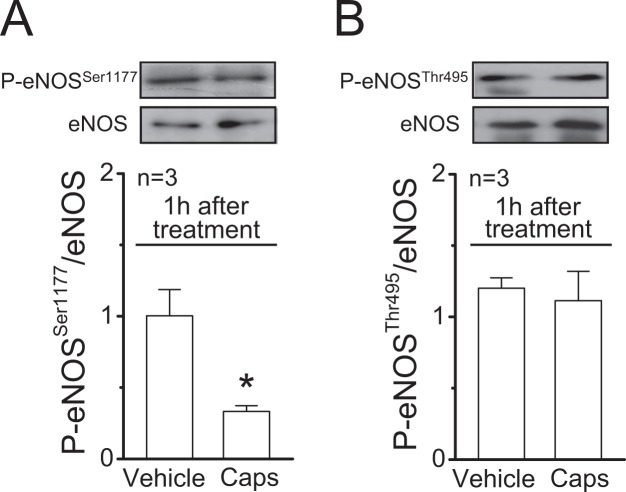
Stimulation of perivascular capsaicin-sensitive sensory nerves leads to a reduction of eNOS phosphorylation at serine 1177. (**A**,**B**) Representative Western blots and densitometric analysis of eNOS phosphorylation at serine 1177 (P-eNOS^Ser1177^, panel (A)) and at threonine 495 (P-eNOS^Thr495^, panel (B)) 1 h after the treatment of mesenteric arteries with 1 µM capsaicin (Caps) or the vehicle for 20 min. Changes in eNOS phosphorylation are expressed as the ratio of phosphorylated protein over total protein. Values are means ± SEM. *P < 0.05 vs vehicle by Student’s unpaired t-test.

**Figure 4:**
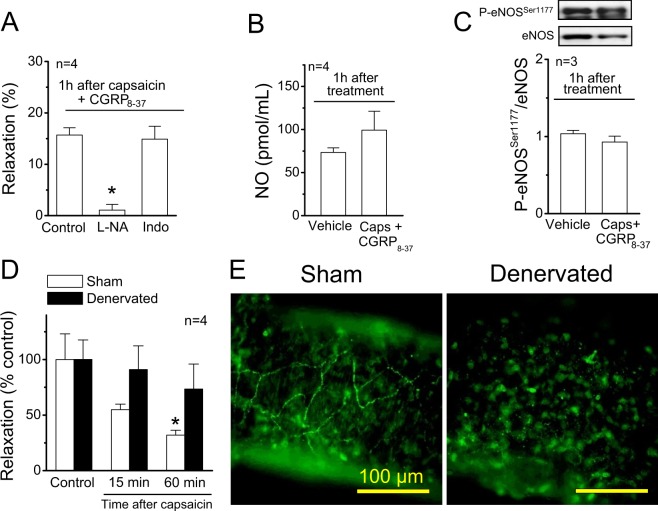
The capsaicin-initiated NO signalling inhibition is mediated by CGRP release from perivascular sensory nerves. (**A**) Relaxation induced by 100 nM ACh in KCl-contracted mesenteric arteries 1 h after capsaicin application (1 µM, 20 min) in the presence of 300 nM CGRP_8–37_. Vasodilator responses were evaluated in control conditions and after the treatment with 100 µM N^G^-nitro-L-arginine (L-NA) or 10 µM indomethacin (Indo). (**B**) NO production induced by ACh (100 nM) observed in mesenteric arterial beds 1 h after the treatment with capsaicin in the presence of 300 nM CGRP_8–37_ or the vehicle. (**C**) Representative Western blots and densitometric analysis of eNOS phosphorylation at serine 1177 (P-eNOS^Ser1177^) in mesenteries treated 1 h before with capsaicin (Caps) in the presence of 300 nM CGRP_8–37_ or the vehicle of capsaicin. (**D**) Relaxation induced by 100 nM ACh in KCl-contracted resistance arteries of sham and denervated mesenteries before (control) and 15 or 60 min after the treatment with capsaicin (1 µM, 20 min). ACh was applied in the presence of 10 µM indomethacin to prevent the interference of prostaglandins in the response, as shown in the experimental protocol depicted in Fig. 2. (**E**) Detection of perivascular sensory nerves through immunofluorescence analysis of CGRP expression. Values are means ± SEM. *P < 0.001 vs control by one-way ANOVA plus Dunnett post hoc test. Scale bars represent 100 µm.

As NO production in endothelial cells is regulated by eNOS phosphorylation, we evaluated the effect of capsaicin on eNOS phosphorylation at serine 1177 (P-eNOS^Ser1177^) and at threonine 495 (P-eNOS^Thr495^). Both P-eNOS^Ser1177^ and P-eNOS^Thr495^ were clearly detected in control conditions (vehicle) and a strong reduction in the level of P-eNOS^Ser1177^, but not in P-eNOS^Thr495^, was observed 1 h after the treatment with capsaicin (Fig. 3A,B). The analysis of total protein showed that the reduction in P-eNOS^Ser1177^ was not associated with a change in eNOS expression (Supplementary Fig. S4). As it has been suggested that capsaicin can modulate eNOS signalling through activation of perivascular sensory nerves^16^, we evaluated if the inhibitory effect triggered by capsaicin was mediated by CGRP release. In line with this notion, blockade of CGRP receptors with 300 nM CGRP_8–37_ prevented the inhibition of the ACh-induced L-NA-sensitive vasodilation (Fig. 4A) and, consistent with this result, capsaicin did not affect the ACh-elicited NO production (Fig. 4B) and the level of P-eNOS^Ser1177^ (Fig. 4C) in the presence of this blocker, which was not associated with a change in eNOS expression (Supplementary Fig. S4). To confirm the participation of perivascular sensory nerves in the response activated by capsaicin, we used denervated mesenteric resistance arteries and sham-operated animals with intact mesenteric innervation, as control. As expected, the vasodilation evoked by capsaicin in intact arteries was absent in denervated preparations (Supplementary Fig. S5A). In addition, although the CGRP-induced vasodilation was similar in denervated and control arteries (Supplementary Fig. S5B), the capsaicin-initiated gradual inhibition of the ACh-induced relaxation was not observed in denervated mesenteries (Fig. 4D), supporting the involvement of CGRP release from perivascular sensory nerves in the inhibitory effect triggered by capsaicin on NO signalling. The elimination of perivascular CGRP-containing sensory nerves was corroborated by immunofluorescence analysis (Fig. 4E).

### Sequential activation of Panx-1 channels and connexin hemichannels in endothelial cells

**Figure 5:**
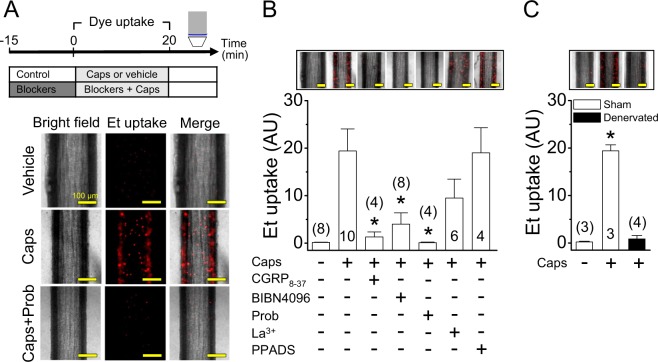
Activation of capsaicin-sensitive sensory nerves leads to CGRP receptor-mediated Panx-1-formed channel opening. (**A**) Experimental protocol and representative images of ethidium (Et) uptake observed during vehicle or 1 µM capsaicin (Caps) application for 20 min in mesenteric resistance arteries in the absence and presence of 1 mM probenecid (Prob), a Panx-1 blocker. (**B**) Representative images and densitometric analysis of Et uptake attained during the stimulation for 20 min with the vehicle of capsaicin or capsaicin in the absence and presence of 300 nM CGRP_8–37_, 10 µM BIBN4096, 1 mM probenecid (Prob), 200 µM La^3+^ and 10 µM PPADS. (**C**) Capsaicin-induced Et uptake observed in control (Sham-operated rats) and denervated mesenteric arteries. Changes in ethidium-fluorescence signal are expressed in arbitrary units (AU). Numbers inside the bars or in parentheses indicate the n value. Values are means ± SEM. *P < 0.05 vs caps (**B**) or vehicle (**C**) by one-way ANOVA plus Dunnett post hoc test. Scale bars represent 100 µm.

CGRP stimulates the opening of Panx-1-formed channels in resistance vessels^16^. Then, we analysed the participation of these channels in the response to capsaicin by assessing ethidium uptake. As expected, the nuclear fluorescent signal of ethidium was barely detected in mesenteric resistance arteries in control conditions (Vehicle, Fig. 5A,B). However, 1 µM capsaicin (20 min) and 100 nM CGRP (5 min) elicited a strong increase in ethidium uptake during the stimulation period (Figs 5A,B and S6A). Consistent with the activation of Panx-1 channels by CGRP, the presence of CGRP_8–37_ or probenecid during the capsaicin treatment fully prevented the increase in ethidium uptake detected at the end of capsaicin application (Fig. 5A,B). The participation of CGRP release from sensory nerves in the response was further confirmed using BIBN4096, a non-peptide CGRP receptor antagonist (Fig. 5B), and denervated arteries (Fig. 5C). Although probenecid completely blocked the increase in ethidium uptake induced by capsaicin, the inhibition of connexin-formed hemichannels with La^3+^ also attenuated the response (Fig. 5B), suggesting the possible sequential activation of these two types of channels. The involvement of ATP release in this process was discarded because blockade of purinergic receptors with pyridoxalphosphate-6-azophenyl-2′,4′-disulfonic acid (PPADS) during the stimulation period did not affect the ethidium uptake attained at the end of capsaicin application (Fig. 5B). Additionally, control experiments confirmed that treatment with either probenecid, La^3+^, CGRP_8–37_, BIBN4096 or PPADS alone did not modify the ethidium uptake observed in basal conditions (data not shown).

**Figure 6:**
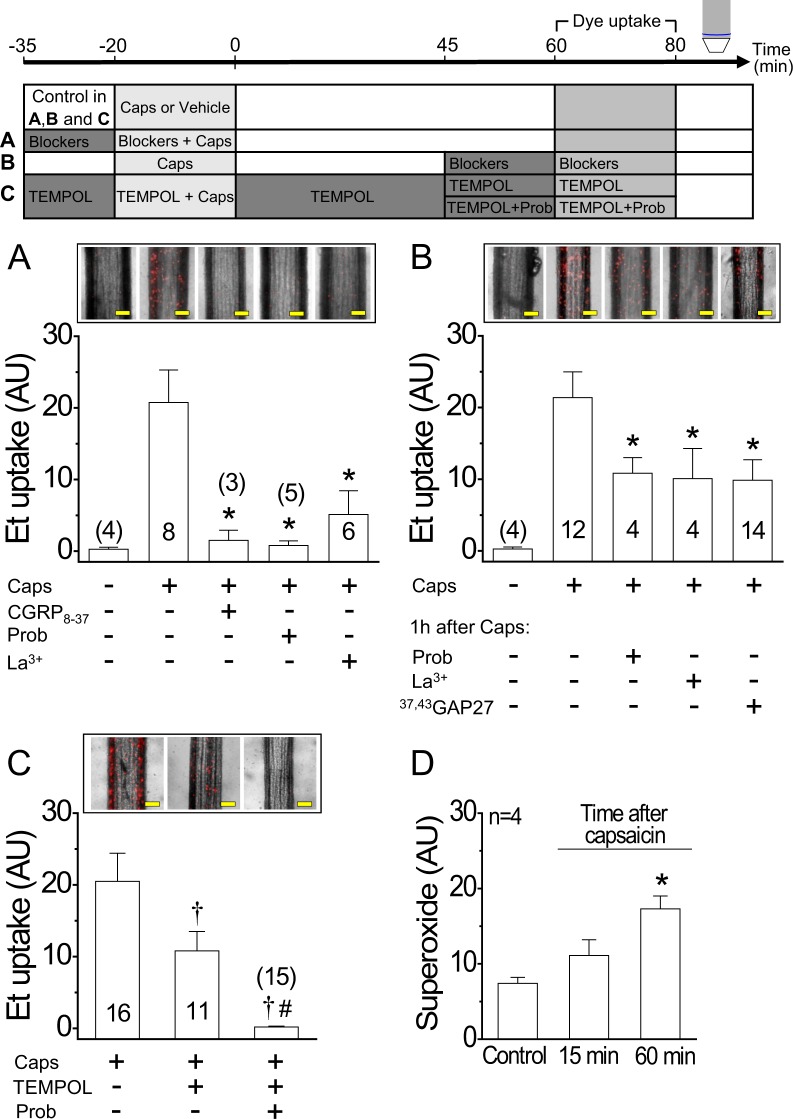
Activation of Panx-1 channels by capsaicin persists along the time and recruits connexin-based hemichannels through a O_2_^• −^-dependent pathway. The experimental protocol of the results shown in A, B and C is depicted on the top of the figure. (**A**) Representative images and densitometric analysis of the ethidium (Et) uptake attained in 20 min. Et uptake was evaluated 1 h after the end of the application of 1 µM capsaicin (Caps, 20 min) in mesenteric resistance arteries. Capsaicin was applied in the absence and presence of 300 nM CGRP_8–37_, 1 mM probenecid (Prob) or 200 µM La^3+^. (**B**) Et uptake observed 1 h after the end of capsaicin application. Et was perfused for 20 min in the presence of 1 mM probenecid (Prob), 200 µM La^3+^ or 200 µM ^37,43^GAP27. (**C**) Et uptake observed 1 h after the end of capsaicin application. As shown in panel B, ethidium was perfused for 20 min, but, in this case, in the presence of TEMPOL alone or in combination with probenecid (Prob). (**D**) O_2_^• −^ production recorded before or 15 and 60 min after capsaicin treatment (1 µM, 20 min). Changes in ethidium-fluorescence signal and O_2_^• −^ formation are expressed in arbitrary units (AU). Numbers inside the bars or in parentheses indicate the n value. Values are means ± SEM. *P < 0.05 vs caps (**A**,**B**) or control (**D**) by one-way ANOVA plus Dunnett post hoc test. ^†^P < 0.05 vs caps and ^#^P < 0.05 vs caps + TEMPOL by one-way ANOVA plus Newman-Keuls post hoc test. Scale bars represent 100 µm.

To assess if the capsaicin-triggered gradual decay of the NO-mediated vasodilation was associated with a persistent opening of Panx-1 channels and connexin hemichannels, we evaluated ethidium uptake 1 h after the end of the capsaicin or CGRP treatment. Interestingly, the increase in ethidium uptake observed at the end of the stimulation with capsaicin or CGRP persisted 1 h later (Figs 6A and S6B) and the presence of CGRP_8–37_, probenecid or La^3+^ during capsaicin application blocked this response (Fig. 6A). The increase in membrane permeability observed 1 h after capsaicin treatment was restricted to small molecules that can diffuse through channels formed by pannexins or connexins, as demonstrated by the lack of uptake of dyes of high molecular weight, such as FITC-dextran (3,000 Da) (Supplementary Fig. S7). Furthermore, to evaluate the contribution of Panx-1 channels and connexin hemichannels to the ethidium uptake recorded 1 h after capsaicin-sensitive sensory nerve stimulation, blockers were applied 1 h after capsaicin treatment, during the assessment of dye uptake. Concomitant application of La^3+^ or probenecid with ethidium attenuated in a similar magnitude the dye uptake observed 1 h after stimulation with capsaicin (Fig. 6B). Notably, treatment with the connexin blocking peptide ^37, 43^GAP27 mimicked the inhibition attained with La^3+^ (Fig. 6B), suggesting that connexin-formed hemichannels were recruited along the time. Noteworthy, a similar reduction in ethidium uptake was observed in the presence of TEMPOL and, in these conditions, further application of probenecid abrogated the response (Fig. 6C), suggesting the involvement of O_2_^• −^ in the recruitment of connexin-formed hemichannels. In line with this hypothesis, a progressive increase of O_2_^• −^ production was detected after the end of capsaicin application (Fig. 6D).

**Figure 7:**
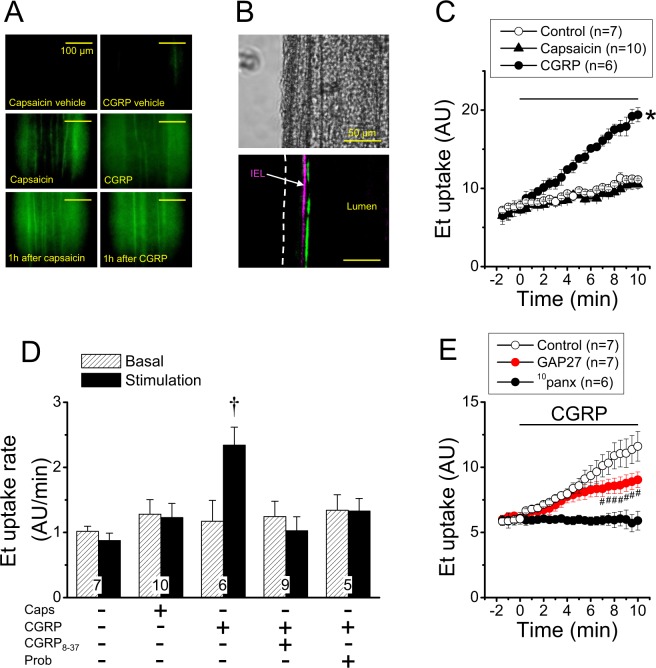
The CGRP-mediated increase in ethidium uptake induced by capsaicin is initiated by Panx-1-formed channel opening in endothelial cells. (**A**) Representative images of the Lucifer Yellow uptake (green) induced by perfusion of 1 µM capsaicin and 100 nM CGRP in mesenteric resistance arteries during the stimulation period (10 min) or 1 h after. (**B**) Representative images of Lucifer Yellow uptake attained after 20 min stimulation of isolated mesenteric resistance arteries with 1 µM capsaicin. In these experiments, Lucifer Yellow and capsaicin were applied in the bath solution. Note that the Lucifer Yellow fluorescent signal is only observed at the inner side of the internal elastic lamina (IEL), confirming that capsaicin activated the uptake of this dye exclusively in the endothelium. (**C**) Time course of the ethidium (Et) uptake achieved in control conditions and after the stimulation with 1 µM capsaicin or 100 nM CGRP in primary cultures of mesenteric endothelial cells. The horizontal bar indicates the period of stimulation. (**D**) Analysis of the Et uptake rate observed during the stimulation with capsaicin (Caps) or CGRP in control conditions and in the presence of 1 µM CGRP_8–37_ or 1 mM probenecid (Prob). The rate of ethidium uptake was assessed by calculating the slope of the increase in fluorescence intensity along the time in basal conditions and during the stimulation period. (**E**) Time course of the ethidium uptake induced by CGRP in the absence and presence of a combination of the connexin blocking peptides ^37,43^GAP27 (200 µM) plus ^40^GAP27 (200 µM) or the Panx-1 blocking peptide ^10^panx (60 µM). Note that, after treatment with GAP 27 peptides, the ethidium uptake curve shows two components: an initial increase similar to control that starts to decline after 6 min. Changes in ethidium-fluorescence signal are expressed in arbitrary units (AU). Numbers inside the bars indicate the n value. Values are means ± SEM. *P < 0.05 vs control by one-way ANOVA plus Dunnett post hoc test. ^†^P < 0.01 vs basal by Student’s paired t-test. ^#^P < 0.05 vs control by two-way ANOVA plus Fisher´s LSD post hoc test.

To identify the cell type involved in the ethidium uptake activated by capsaicin or CGRP, we used Lucifer yellow (LY), because this dye stains the whole cell and does not diffuse through myoendothelial gap junctions^34^. The dye uptake attained after perfusion of LY in control conditions was not apparent (Fig. 7A), but in contrast, capsaicin or CGRP stimulation resulted in a strong increase in LY fluorescent signal in endothelial cells (Fig. 7A), which is evidenced by the orientation of these cells along to the longitudinal axis of the vessel. In addition, a similar pattern of LY uptake was observed 1 h after capsaicin or CGRP stimulation (Fig. 7A). As luminal application of LY may favour its uptake by endothelial cells, we also evaluated the response evoked by capsaicin in resistance arteries in which LY was applied in the bath solution during the stimulation period. In these conditions, the fluorescent signal was only evident at the luminal side of the internal elastic lamina (IEL), corroborating that the capsaicin-activated LY uptake is restricted to the endothelium (Fig. 7B). To confirm that the response was triggered in the endothelium by a CGRP receptor-mediated Panx-1 channel opening, we used primary cultures of mesenteric endothelial cells. In these cells, CGRP induced an increase in ethidium uptake that was sensitive to CGRP_8–37_ and probenecid (Fig. 7C,D), as observed in intact mesenteric arteries. In contrast to CGRP, capsaicin did not affect cell membrane permeability to ethidium (Fig. 7C,D). Interestingly, the time course of CGRP-induced ethidium uptake showed two components after the inhibition of connexin hemichannels with a combination of ^37, 43^GAP27 and ^40^GAP27: an initial component that was similar to the control curve, which decayed after 6 min of the CGRP stimulation period to stabilize in a secondary blunted component (Fig. 7E). As the treatment with ^10^panx blocked the response from the beginning of the stimulation (Fig. 7E), in conjunction, these results support the notion that connexin hemichannels are recruited after CGRP-mediated Panx-1 channel opening.

### Panx-1 channels and connexin hemichannels mediate the NO signalling inhibition

**Figure 8:**
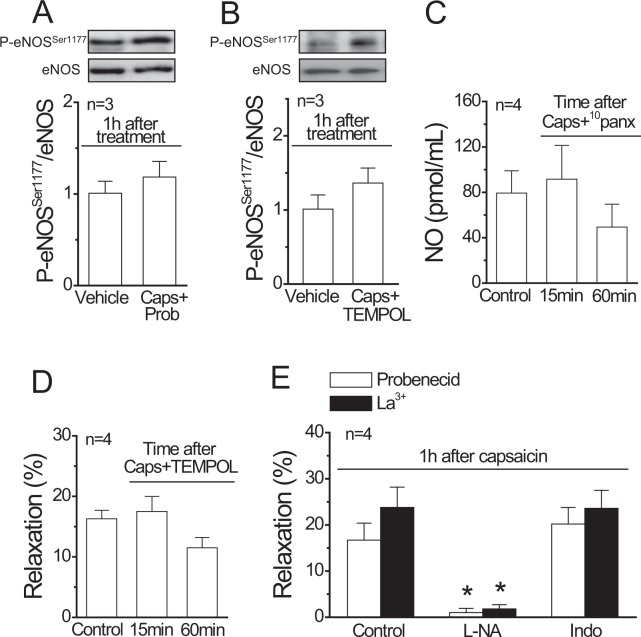
Participation of Panx-1 channels, connexin hemichannels and O_2_^• −^ production in the progressive inhibition of the NO-dependent relaxation triggered by capsaicin-sensitive perivascular sensory nerve activation. (**A**,**B**) Representative Western blots and densitometric analysis of eNOS phosphorylation at serine 1177 (P-eNOS^Ser1177^) in mesenteries 1 h after the treatment with the vehicle of capsaicin or capsaicin (Caps) in the presence of probenecid (Prob, **A**) or TEMPOL (**B**). (**C**) ACh (100 nM)-induced NO production in mesenteric arterial beds before (control) and 15 or 60 min after capsaicin application in the presence of the Panx-1 blocking peptide ^10^panx (60 µM). (**D**) Relaxation induced by 100 nM ACh in KCl-contracted resistance arteries observed in the presence of TEMPOL before (control) and 15 or 60 min after the treatment with capsaicin (1 µM, 20 min). The treatment with TEMPOL started 15 min before the stimulation with ACh in control conditions and was maintained until the end of the experiment. (**E**) Relaxation induced by 100 nM ACh in KCl-contracted mesenteric arteries 1 h after 1 µM capsaicin application (20 min) in the presence of 1 mM probenecid or 200 µM La^3+^. The relaxation was evaluated in control conditions and in the presence of 100 µM N^G^-nitro-L-arginine (L-NA) or 10 µM indomethacin (Indo). Values are means ± SEM. *P < 0.05 vs control by one-way ANOVA plus Dunnett post hoc test.

The long lasting (>1 h) activation of Panx-1 channels and further O_2_^• −^-mediated recruitment of connexin hemichannels may lead to endothelial dysfunction. Consistent with this hypothesis, application of probenecid or TEMPOL during sensory nerve stimulation with capsaicin prevented the reduction in P-eNOS^Ser1177^ (Fig. 8A,B) and, likewise, the time-dependent decrease of the ACh-induced NO production and subsequent vasodilation (Fig. 2) was not observed in the presence of ^10^panx and TEMPOL, respectively (Fig. 8C,D). In line with these results, application of La^3+^ or probenecid during capsaicin treatment prevented the inhibition of NO-dependent vasodilation, since, in these conditions, the response induced by ACh 1 h after capsaicin application was abolished by L-NA and was insensitive to indomethacin (Fig. 8E).

## Discussion

**Figure 9:**
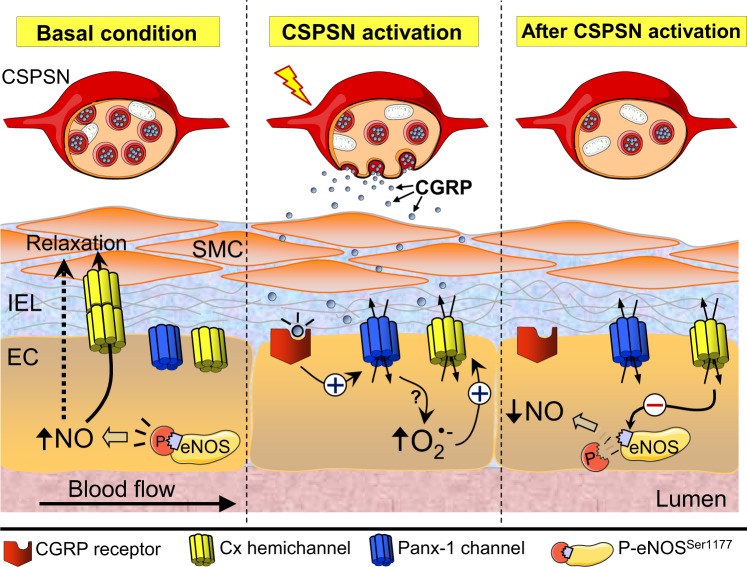
Schematic model of the inhibition of NO signalling evoked by activation of capsaicin-sensitive perivascular sensory nerves. Control of vasomotor tone in resistance arteries relies on the functional interaction between endothelial cells (EC), smooth muscle cells (SMC) and perivascular nerves, including the capsaicin-sensitive perivascular sensory nerves (CSPSN). Although ECs and SMCs are physically separated by the internal elastic lamina (IEL), vasomotor signals generated by ECs, such as nitric oxide (NO), can reach SMCs either diffusing through the IEL or directly via myoendothelial gap junctions. In resistance arteries, phosphorylation of eNOS at serine 1177 (P-eNOS^Ser1177^) contributes to both the basal control of vasomotor tone and the response induced by endothelium-dependent vasodilators. CGRP release during CSPSN stimulation activates CGRP receptors in ECs, which leads to the opening of pannexin-1 (Panx-1)-formed channels and the further O_2_^• −^ formation. In turn, the pannexin-1 channel-triggered increase in O_2_^• −^ causes a long lasting activation of connexin (Cx)-formed hemichannels that provokes a reduction in eNOS phosphorylation at serine 1177, with the consequent decrease in NO production.

NO plays a central role in the control of vasomotor tone in resistance vessels, and then, blood flow distribution and peripheral vascular resistance depends on the fine regulation of eNOS activity. Beside endothelial cells, perivascular sensory nerves are also involved in the control of vasomotor tone by the release of CGRP and substance P. These neuropeptides are potent vasodilators, but, in addition, CGRP have also been shown to participate in the control of eNOS expression in resistance vessels^16^, which suggests that perivascular sensory nerves may be involved in the regulation of NO production. In this study, we analysed the possible participation of capsaicin-sensitive perivascular sensory nerves in the modulation of endothelium-dependent NO-mediated vasodilation and the mechanisms involved in this process. Our results indicate that transient perivascular sensory nerve activation with capsaicin triggers a progressive inhibition of the NO-mediated vasodilator response evoked by ACh through a CGRP receptor-initiated pathway. The progressive detriment of this response was associated with a reduction in NO production triggered by the opening of Panx-1 channels and the subsequent O_2_^• −^-dependent activation of connexin hemichannels in the endothelium, which persists along the time and leads to a reduction in P-eNOS^Ser1177^ levels (Fig. 9).

Although prostaglandins, such as prostacyclin, have been proposed to contribute to the endothelium-dependent vasodilation, the relaxation induced by ACh in resistance arteries has typically been observed to depend only on NO and EDHF^35,36^. Consistent with this notion, treatment with indomethacin did not affect the vasodilator response activated by ACh in control conditions (Figs 1 and S1). Furthermore, blockade of NO production with L-NA either attenuated or abolished the response to ACh in resistance arteries contracted with PE or KCl, respectively (Fig. 1), and in both cases (PE or KCl), the magnitude of the NO-dependent vasodilator component was similar, which supports the concept that the L-NA-resistant vasodilation is mediated by a prostaglandin-independent EDHF signal. Interestingly, transient stimulation with capsaicin (20 min) resulted in a time-dependent reduction of NO production and the consequent NO-dependent vasodilator component activated by ACh (Figs 1 and 2), which was evident from 15 min after the end of the capsaicin treatment and continued gradually developing, for at least 45 min more, to reach almost the complete inhibition of ACh-initiated NO production. Despite of this inhibition, capsaicin did not affect the magnitude of the vasodilation induced by ACh, because the NO-mediated vasodilator component was replaced by an indomethacin-sensitive vasodilation (Fig. 1), probably evoked by prostaglandins. The upsurge of this indomethacin-sensitive mechanism may have masked the effect of capsaicin on the response to ACh described in previous works, in which capsaicin was proposed to disrupt an endothelium-independent vasodilator component activated by high concentrations of ACh, but, in contrast, did not affect the endothelium-dependent response analysed in the present study^37^. Additionally, it should be noted that the contribution of prostaglandins or NO production to the ACh-induced vasodilation observed after capsaicin application has not been evaluated previously.

The magnitude of the L-NA-resistant response induced by ACh was quite similar to that observed in the presence of indomethacin after the treatment with capsaicin, which suggests that capsaicin triggered an inhibitory signalling cascade that specifically targeted NO production, without altering the EDHF-mediated vasodilator pathway. As EDHF may modulate eNOS activation^35,38^, to further analyse the inhibition of NO production by capsaicin, we used KCl-contracted mesenteries to disable the EDHF signalling. In these conditions, capsaicin also led to a gradual decay along the time of the NO-dependent vasodilation induced by ACh, which was confirmed by direct NO measurements and the demonstration that capsaicin did not affect the relaxing response induced by NO directly on smooth muscle cells using the NO donor SNAP (Fig. 2). In addition, the sensitivity to indomethacin observed 1 h after capsaicin treatment (Fig. 1) shows that the decline in NO signalling was followed by the emergence of a prostaglandin-dependent vasodilator component, probably, prostacyclin, which may reflect that NO interferes with prostaglandin production. However, it should be noted that the indomethacin-sensitive vasodilation did not arise in parallel to the reduction of NO signalling, since it was not apparent 15 min after capsaicin treatment (Supplementary Fig. S2), which suggests that the generation of prostaglandins was only enabled once NO production reached low levels. Consistent with this hypothesis, NO has been shown to restrain the prostaglandin signalling^39^ and our data show that prolonged inhibition of NO production (>1 h) uncovers an indomethacin-sensitive vasodilator component (Supplementary Fig. S2) that was absent in control conditions or after NOS blockade for shorter periods of time (Figs 1 and S1). In this context, it is important to note that eNOS inhibition in resistance arteries, with blockers such as L-NA, is a slow, time-dependent process that takes longer than 45 min to attain the maximal effect^40^. Furthermore, perivascular sensory nerves activation has been reported to enhance prostacyclin production through CGRP release^41,42^, although the possible participation of NO inhibition in this process has not been evaluated.

We recently showed that activation of capsaicin-sensitive perivascular sensory nerves of mesenteric arteries leads to a slow reduction in eNOS expression that reached statistical significance 3 h after the end of capsaicin stimulation^16^. However, the decrease in the ACh-induced NO-mediated vasodilation observed in the present work was much faster, since it was detected 15 min after the end of capsaicin application (Fig. 2). NO production is modulated through eNOS phosphorylation, which provides a rapid control mechanism of NO signalling^43^. The most well-characterized eNOS phosphorylation sites are P-eNOS^Ser1177^ and P-eNOS^Thr495^ ^44,45^. Whereas P-eNOS^Ser1177^ enhances Ca^2+^-mediated eNOS activation^46^, P-eNOS^Thr495^ reduces eNOS activity by decreasing the Ca^2+^-sensitivity of the enzyme^47^. Although capsaicin did not trigger an increase in P-eNOS^Thr495^, as may have been expected, it elicited a reduction in P-eNOS^Ser1177^ (Fig. 3). The response to ACh relies on an increment in intracellular Ca^2+^ concentration^35^, and then, the reduction in P-eNOS^Ser1177^ certainly contributes to the inhibition of the ACh-induced NO production, but the direct involvement of an alteration in the intracellular Ca^2+^ signalling cannot be ruled out.

Capsaicin is an agonist of TRPV1 receptors, which are selectively expressed in a subpopulation of perivascular sensory nerves^6^. TRPV1 activation leads to sensory afferent fibres depolarization, and thereby, neurotransmitter release. CGRP and substance P are the most relevant neuropeptides released by capsaicin-sensitive perivascular sensory nerves^48^ and, as CGRP is a potent vasodilator, this neuropeptide has been proposed to be involved in the control of resistance vessel function. Consistent with the participation of CGRP in the response to capsaicin, the inhibition of ACh-induced NO-dependent vasodilation was not observed in denervated arteries or in the presence of CGRP_8–37_, an antagonist of CGRP receptors. In addition, blockade of CGRP receptors also prevented the reduction in P-eNOS^Ser1177^ and the inhibition of ACh-induced NO production (Fig. 4), which confirms that the capsaicin-initiated inhibition of NO signalling was mediated by CGRP release from sensory nerves and the subsequent CGRP receptor activation in the vascular wall (Fig. 9).

In addition to be a vasodilator, CGRP has been reported to evoke an increase in plasma membrane permeability to dyes of small molecular weight, such as ethidium, by inducing the Panx-1 channel opening in the wall of mesenteric arteries through a CGRP_8–37_-sensitive pathway, which, in contrast, was not observed in response to substance P^16^. In this study, we extended these results by showing that capsaicin also induced a strong increment in ethidium or LY uptake in endothelial cells of mesenteric resistance arteries. This response was fully prevented by CGRP_8–37_, BIBN4096, probenecid or the Panx-1 blocking peptide ^10^panx (Figs 5 to 7), indicating that CGRP released from capsaicin-sensitive perivascular sensory nerves activates a CGRP receptor-mediated opening of Panx-1-formed channels in mesenteric resistance arteries. CGRP receptors as well as Panx-1 are expressed in endothelial and smooth muscle cells of resistance arteries^30,49^, but, apparently, CGRP released in response to capsaicin evokes the opening of Panx-1 channels exclusively in endothelial cells, because the uptake of LY induced by capsaicin or CGRP was restricted to the endothelium and CGRP activated a probenecid and ^10^panx-sensitive increase in ethidium uptake in cultured mesenteric endothelial cells (Fig. 7). Although TRPV-1 channels have been suggested to be also expressed in endothelial cells^50,51^, capsaicin did not induce an increase in ethidium uptake in denervated arteries (Fig. 5) or in cultured endothelial cells (Fig. 7), which is consistent with the notion that capsaicin depends on CGRP release from perivascular sensory nerves to induce the opening of Panx-1 channels in endothelial cells (Fig. 9).

In addition to Panx-1 channels, our results indicate that connexin-formed hemichannels are also involved in the response, because treatment with La^3+^ attenuated the capsaicin-triggered ethidium uptake (Figs 5 and 6). Interestingly, the inhibition of Panx-1 channels abolished the response (Fig. 5), suggesting that the activation of connexin hemichannels occurs downstream from Panx-1 channel opening. In agreement with this notion, the analysis of the time course of the CGRP-induced dye uptake in cultured endothelial cells showed that blockade of connexin-formed hemichannels with the connexin blocking peptides ^37, 43^Gap27 and ^40^Gap27 only inhibited the increase in ethidium uptake after several minutes of stimulation, revealing a secondary component of the response that depended on the initial activation of Panx-1-formed channels (Fig. 7). Panx-1 channels may lead to the activation of connexin hemichannels through an ATP release-mediated pathway^52^; however, the inhibition of purinergic receptors with PPADS did not affect the ethidium uptake observed at the end of capsaicin treatment (Fig. 5), suggesting that this mechanism is not involved in the capsaicin-initiated sequential activation of Panx-1 channels and connexin hemichannels in mesenteric resistance vessels.

The opening of Panx-1 channels and connexin hemichannels was temporally related with the gradual decay of the NO-dependent vasodilation, because the capsaicin- and CGRP-activated ethidium uptake was prolonged for at least 1 h. This increase in membrane permeability observed 1 h after capsaicin or CGRP application was not the result of cell damage, since the dye of high molecular weight, FITC-dextran (3,000 Da), was only able to enter into endothelial cells that were previously permeabilized by perfusion of Triton X-100 (Supplementary Fig. S7). In this context, it is important to note that this sustained response relied on both the initial opening of Panx-1 channels and the further activation of connexin hemichannels (Fig. 6), which indicates that the activity of both channels is required to maintain the response. Consistent with this notion, the application of the blocking peptides ^10^panx or ^37,43^Gap 27 1 h after the end of the stimulation with capsaicin, during the assessment of ethidium uptake, showed that both channels contribute in a similar magnitude to the sustained increase in cell membrane permeability observed at that time (Fig. 6). These data also indicate that the hemichannels involved in the response are formed by Cx37, Cx43 or both.

It has been shown that NADPH oxidase-derived O_2_^• −^ is involved in the vasodilator response initiated by capsaicin through the release of CGRP and substance P^53^ and oxidative stress has been related to the opening of Cx43-formed hemichannels^54^; then, we hypothesized that reactive oxygen species may be involved in the capsaicin-evoked responses. Interestingly, the development of endothelial dysfunction was paralleled by an increase in O_2_^• −^ production and the connexin-dependent component of ethidium uptake was not observed in the presence of TEMPOL, a scavenger of O_2_^• −^ (Fig. 6), which suggests that a O_2_^• −^-dependent signal pathway participates in the recruitment of connexin hemichannels (Fig. 9).

Panx-1 channels and connexin hemichannels allow the passage of ions and molecules that are important to keep cell homeostasis, and then, prolonged opening of these channels may affect the ion electrochemical gradient and may lead to Ca^2+^ overload^55^. Therefore, we hypothesized that the prolonged increase in the activation of Panx-1 channels and connexin hemichannels may trigger endothelial dysfunction and the inhibition of NO signalling evoked by capsaicin. Consistent with this hypothesis, blockade of Panx-1 channels with probenecid or connexin hemichannels with La^3+^ fully prevented the inhibition of the NO-dependent vasodilation induced by ACh 1 h after capsaicin-sensitive sensory nerve activation (Fig. 8). In line with these results, probenecid and ^10^panx also prevented the capsaicin-triggered reduction in P-eNOS^Ser1177^ levels and NO production, respectively (Fig. 8). In addition, the gradual inhibition of the NO-dependent vasodilation and P-eNOS^Ser1177^ levels were not evident in intact vessels treated with TEMPOL (Fig. 8), which support our findings and is coherent with the hypothesis that CGRP release from perivascular sensory nerves mediates the response to capsaicin by leading to the opening of Panx-1 channels and the further activation of Cx37 or Cx43 hemichannels through a O_2_^• −^-dependent mechanism (Fig. 9).

The physiological role of the CGRP released by perivascular sensory nerves has not been clearly defined^56^. Although CGRP can be detected in plasma, which indicates that this neurotransmitter is tonically released in physiological conditions, deletion of CGRP or intravenous application of CGRP receptor antagonists did not affect mean arterial pressure^9,57^. Nevertheless, the potent vasodilator activity of CGRP has led to propose that it plays a protective role in the cardiovascular system^9,56^. In this context, CGRP expression was found to be increased in angiotensin II-elicited hypertension and in pressure overload heart failure induced by transverse aortic constriction^58,59^. In addition, the vascular hypertrophy and fibrosis observed in these models of hypertension and heart failure were enhanced in CGRP knockout animals^58,59^. However, on the other hand, pro-inflammatory stimuli evoke a strong CGRP release from perivascular sensory nerves that leads to an important increase in the plasma concentration of this peptide^11,12^. Furthermore, the development of inflammatory responses is associated with the activation of Panx-1 channels and connexin hemichannels, which has been proposed to contribute to cell damage and dysfunction characteristic of this pathological condition^31,52^. Therefore, in the context of our results, the activation of perivascular sensory nerves and CGRP release may be involved in the reduction of the NO signalling associated to the development of the endothelial dysfunction typically observed in pro-inflammatory events such as ischemia and reperfusion or septic shock^60,61^. Interestingly, the endothelial dysfunction triggered by a transient pro-inflammatory event persists along the time^60,62^, which is consistent with the temporal characteristics of the CGRP-elicited NO production inhibition.

In summary, our data indicate that transient activation of capsaicin-sensitive perivascular sensory nerves leads to the inhibition of ACh-induced NO-dependent vasodilation through the release of CGRP. The CGRP receptor signalling in endothelial cells of resistance arteries triggers the activation of Panx-1 channels and the further O_2_^• −^-dependent connexin hemichannel opening, which results in a sustained increase in plasma membrane permeability along the time that initiates a decline in NO production, at least in part, by the reduction in P-eNOS^Ser1177^ levels observed in resting conditions (Fig. 9). These results suggest that CGRP receptors, Panx-1 channels and connexin hemichannels are potential targets for the design of novel pharmacological strategies for the treatment of the complications related to the NO deficiency and endothelial dysfunction associated to inflammation.

## Methods

Male Sprague-Dawley rats (200–230 g) were bred and maintained at the Research Animal Facility of Pontificia Universidad Católica de Chile. All experimental procedures were approved by the Institutional Bioethics Committee and the experiments were conducted according to the Helsinki Declaration.

### Perfusion of isolated mesenteric arterial bed

Rats were anesthetized with xylazine and ketamine (10 and 90 mg/Kg i.p., respectively) and the isolated mesenteric arterial bed was prepared, as described previously^63^. The abdomen of anesthetized rats was open by a midline incision and the superior mesenteric artery was cleared and cannulated in order to perfuse the mesentery with a Tyrode buffer solution (in mM: NaCl 118; KCl 5.4; CaCl_2_ 2.5; KH_2_PO_4_ 1.2; MgSO_4_ 1.2; glucose 11.1 and NaHCO_3_ 23.8) kept at 37 °C and equilibrated with 5% CO_2_–95% O_2_, to yield a pH 7.35–7.45. The perfusion flow rate was set at 2 ml/min using a peristaltic pump (Peri-Star, WPI) and mesenteries were severed from the intestinal wall and placed in a perfusion chamber. Therefore, after this procedure, only the mesenteric arterial bed is perfused in this preparation. The aorta was cut to euthanize the animals by exsanguination under deep anaesthesia. Experiments were started after the preparation was allowed to stabilize for 20 min. Changes in perfusion pressure were detected using a pressure transducer (P23Db Statham) connected at the entrance of the superior mesenteric artery and were recorded using the WinDaq software (DataQ instruments Inc., USA). All drugs were applied dissolved in the perfusion solution.

### Denervation of mesenteric arteries

Mesenteric arteries were denervated as described by Hobara *et al*.^64^. Briefly, the superior mesenteric artery of anesthetized rats (200 g) was cleared of connective tissue through a small incision in the abdominal wall and a drop of 10% phenol/90% ethanol solution or a sterile saline solution was applied on the vessel wall surface for 20 min to damage perivascular nerves (denervated arteries) or as control (sham-operated rats), respectively. The incision was sutured and, seven days later, the mesenteric arterial bed was prepared as described above or fixed for immunofluorescence analysis.

### Isolation of microvascular mesenteric endothelial cells

Endothelial cells were isolated as described by Ashley *et al*.^65^. Briefly, isolated mesenteric beds were perfused for 5 min with a sterile Tyrode buffer solution containing a mixture of antibiotics and antimycotics (Anti-Anti solution, Gibco, Invitrogen, NY, USA). Then, mesenteric vessels were digested with a physiological saline solution containing 0.2% collagenase type I and 0.1% BSA for 1 h at 37 °C. The resulting suspension was rinsed with M-199 media and centrifuged. Pelleted cells were resuspended in M-199 media, centrifuged and resuspended again in M-199 media containing 20% fetal bovine serum (FBS) and 0.002% endothelial cell growth supplement from bovine pituitary (ECGS). Finally, cells were seeded directly onto sterile glass coverslips. Four hours later, non-adherent cells were removed, and the remaining adherent endothelial cells were kept at 37 °C in a 5% CO_2_–95% air atmosphere at nearly 100% relative humidity. After two days, microvascular endothelial cells reached 70 to 80% of confluence and the culture media was replaced by a MOPS-buffered Tyrode solution (pH 7.4, bicarbonate was omitted) to perform the experiments.

### Detection of NO production

NO was detected using a NO sensor electrode (AmiNO-700) and the inNO-T-II measuring system (Innovative instruments Inc., USA). Samples collected from the mesenteric outflow (100 µl) were injected into a solution (20 mL) containing 0.1 M H_2_SO_4_ and 30 mM KI to reduce nitrites to NO. Changes in NO concentration were recorded using the data acquisition software InNOII v2.2 (Innovative instruments Inc., USA). The NO electrode was calibrated at the end of each experiment with 0.1–1000 nM sodium nitrite.

### O_2_^• −^ quantification

Changes in O_2_^• −^ were evaluated using the analysis by emitted light (ABEL^®^) assay. This method is based on the oxidation of pholasin, the photoprotein responsible for luminescence in the bivalve Pholas dactylus. To perform the O_2_^• −^ measurements, mesenteries were prepared as described above and 20 µL of perfusate samples were collected and mixed with 100 µL of adjuvant-K^TM^ solution. After one minute of equilibration, the reaction was initiated by the injection of 250 µL of pholasin solution (10 µg/mL). Pholasin emits an intense luminescence upon oxidation by O_2_^• −^ radicals, which was immediately measured on a Turner TD20e luminometer (Promega). Adjuvant-K^TM^ and pholasin solutions were prepared using the reconstitution buffer (Hank’s balanced salt solution with 20 mM HEPES, pH 7.4) as indicated by the manufacturer (Knight Scientific Ltd.). All measurements were made in duplicate.

### Activity of pannexin channels and connexin hemichannels

Opening of pannexin channels or connexin hemichannels was evaluated by measuring LY or ethidium uptake, as described previously^66^. LY (exciter: 470–490 nm; emission: 515 nm, long pass filter) diffuses through the cytoplasm and stain the whole cell, whereas ethidium (exciter: 530–550 nm; emission: 590 nm, long pass filter) produces nuclear fluorescence after intercalating with DNA. Mesenteric arterial beds were perfused with 30 µM LY or 5 µM ethidium bromide for 5–20 min and, after 15 min of washout, a small resistance artery (120–180 µm inner diameter, ~0.8 cm length) was isolated and pinned down on a Sylgard® (Dow Corning Corporation, MI, USA) surface at the bottom of a 35 mm dish containing MOPS-buffered Tyrode solution (pH 7.4). LY uptake was also evaluated in isolated resistance arteries. In these experiments, the vessel wall was analysed first to record the IEL autofluorescence, and thus, LY was applied simultaneously with 1 µM capsaicin for 20 min in the bath solution. At the end of the stimulation period, LY and capsaicin were removed and the fluorescent signal of the dye was examined. In the case of cultured cells, endothelial cells plated on glass cover slips were incubated in MOPS-buffered Tyrode solution containing 5 µM ethidium bromide and the ethidium fluorescence signal was recorded over time. The epifluorescence was analysed using an intensified CCD camera (Retiga Fast 1394, Q Imaging) and the ImageJ software. Dye uptake was evaluated during or 1 h after capsaicin or CGRP application.

### Western blotting

Mesenteries were homogenized and proteins were separated by 12% SDS-PAGE and transferred onto a PVDF membrane (Pierce, Rockford, IL, USA). The Signal Enhancer HIKARI (Nacalai Tesque, INC, Japan) was used to incubate the primary (BD-Transduction Labs, Lexington, KY, USA) and secondary antibodies (Pierce, Rockford, IL, USA) and SuperSignal® West Femto (Pierce, Rockford, IL, USA) to detect the protein bands. Molecular mass was estimated with pre-stained markers (BioRad, Hercules, CA, USA). Blots were developed for eNOS phosphorylation at serine 1177 (P-eNOS^Ser1177^) or at threonine 495 (P-eNOS^Thr495^), and then, stripped and re-probed for total eNOS and ß-actin. Protein bands were analysed using the ImageJ software and changes in eNOS phosphorylation and total eNOS were expressed as the ratio of phosphorylated protein over total protein and eNOS over ß-actin, respectively.

### Immunofluorescence analysis of intact vessels

The mesenteric arterial bed was perfused with Bouin’s solution to fix the mesenteric arteries. Second order arteries were isolated, permeabilized with 0.01% Triton X-100 and blocked with 3% BSA, as described previously^67^. Vessels were incubated overnight at 4 °C with an anti-CGRP primary antibody (Thermo Scientific, Rockford, IL, USA), and then, with an Alexa-568-labeled goat anti-mouse secondary antibody (Molecular Probes, Eugene, OR, USA) for 4 h at 4 °C. The fluorescent signal was examined with an Olympus BX41 WI microscope and a CCD camera (Jenoptik ProgRes C5).

### Experimental protocols

#### Transient perivascular sensory nerve activation or direct CGRP stimulation

Mesenteric arteries were treated with 1 µM capsaicin for 20 min to activate perivascular sensory nerves or directly with 100 nM CGRP for 5 min. In additional experiments, cultured endothelial cells were stimulated with 1 µM capsaicin or 100 nM CGRP for 10 min. Vasodilator responses, NO production and O_2_^• −^ formation were measured in control conditions and 15 min or 1 h after the end of the treatment with capsaicin. The effect of the vehicle of capsaicin and CGRP was also assessed as control.

### Vasomotor responses

As vessels of the isolated mesentery do not develop myogenic vasomotor tone, the mesenteric arterial bed was constricted with 60 µM PE or 70 mM KCl to be able to assess the vasodilation induced by stimulation with 100 nM ACh for 1 min or 300 nM SNAP for 2 min. High-KCl Tyrode buffer solutions were prepared by equimolar substitution of Na^+^ ions for K^+^ ions. Results were expressed as percentage of reduction in the PE or KCl-induced increase in perfusion pressure (% relaxation), as percentage of change in perfusion pressure along the time (% baseline), or as percentage of the response observed in control conditions (% control).

### Blockers

The effect of the following blockers was examined: 100 µM L-NA, a NOS blocker; 10 µM indomethacin, a cyclooxygenase inhibitor; 0.3–1 µM CGRP_8–37_, a CGRP receptor antagonist; 1 mM probenecid, a Panx-1 channel inhibitor; 200 µM La^3+^, a connexin hemichannel blocker; 10 µM PPADS, a purinergic receptor blocker; 200 µM ^37,43^GAP27, a mimetic peptide of the second extracellular loop of Cx37 and Cx43 designed to inhibit the channels formed by these connexins; 10 µM TEMPOL, a superoxide dismutase mimetic; 60 µM ^10^panx, a Panx-1 channel blocking peptide and 200 µM ^40^GAP27, a Cx40 blocking mimetic peptide. In addition to CGRP_8–37_, the non-peptide CGRP receptor antagonist BIBN4096 was also used and the effective concentration of this blocker in intact vessels was determined by testing the inhibition of the CGRP-induced vasodilation (Supplementary Fig. S8). CGRP_8–37_, probenecid, BIBN4096, La^3+^, PPADS, ^37,43^GAP27, TEMPOL, ^10^panx and ^40^GAP27 were applied from 15 min before the capsaicin/vehicle treatment or the dye uptake analysis. In addition, vessels were treated with L-NA for 45 min or with indomethacin for 15 min, but, in an additional experimental series, L-NA was also applied for 75 min to evaluate the effect of NOS blockade for longer periods of time on the indomethacin-sensitive vasodilator component of the response to ACh.

### Chemicals

Capsaicin, L-NA, indomethacin, MOPS, ethidium bromide, ECGS, FITC-dextran (3000 Da), BSA, PE, ACh, LaCl_3_ and probenecid were purchased from Sigma-Aldrich (St. Louis, MO, USA). PPADS and BIBN4096 were obtained from Tocris Bioscience (Ellisville, MO, USA), SNAP and TEMPOL from Calbiochem (La Jolla, CA, USA), Lucifer Yellow (LY) from Molecular Probes (Eugene, OR, USA), CGRP (alpha isoform, rat) from Bachem (Torrance, CA, USA) and collagenase type I from Worthington (Lakewood, NJ, USA). CGRP_8–37_, ^10^panx, ^37,43^GAP27 and ^40^GAP27 were synthesized by Genscript (Israel). Capsaicin was dissolved in ethanol; indomethacin, BIBN4096 and SNAP in dimethyl sulfoxide (DMSO) and probenecid in 0.5 M NaOH. Then, these drugs were diluted in the buffer solution to the final working concentration. Application of the vehicle of these drugs (ethanol, DMSO or NaOH) did not have effect per se (data not shown).

### Statistical analysis

Values are presented as mean ± SEM. Comparisons between groups were made using paired or unpaired Student´s t-test, one-way ANOVA plus Dunnett or Newman-Keuls post hoc test, or two-way ANOVA plus Fisher´s LSD post hoc test as appropriate. P < 0.05 was considered significant.

## Supplementary information

Supplementary Figures
