# Supplementary material for: CGRP signalling inhibits NO production through pannexin-1 channel activation in endothelial cells
Source: Sci Rep. 2019 May 28;9:7932. doi: 10.1038/s41598-019-44333-w (PMC6538758; doi:10.1038/s41598-019-44333-w)
Supplement: Supplementary file 1 — Supplementary Figures [file 41598_2019_44333_MOESM1_ESM.pdf]

SUPPLEMENTARY FIGURES

CGRP signalling inhibits NO production through pannexin-1 channel activation in endothelial cells

Pablo S. Gaete; Mauricio A. Lillo; Mariela Puebla; Inés Poblete; Xavier F. Figueroa*

Departamento de Fisiología, Facultad de Ciencias Biológicas, Pontificia Universidad Católica de Chile, Santiago 8330025, Chile.

***Author for correspondence:**

Dr. Xavier F. Figueroa

Departamento de Fisiología

Facultad de Ciencias Biológicas

Pontificia Universidad Católica de Chile

Casilla 114-D, Santiago, Chile

Phone: 562-2686-2356

Fax: 562-2354-1850

E-mail: xfigueroa@bio.puc.cl

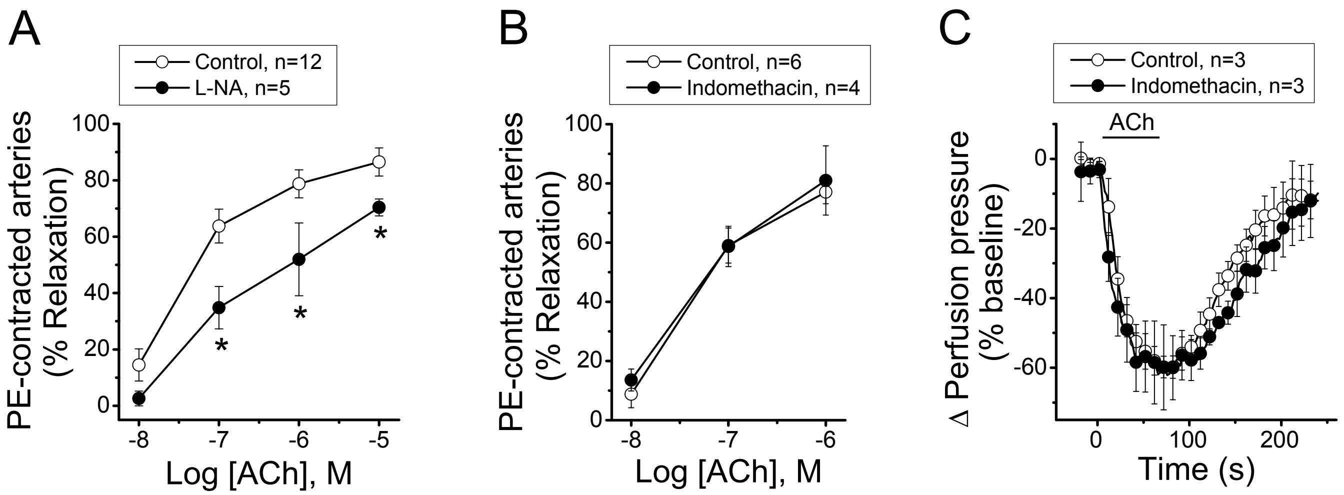

Supplementary Figure S1. Contribution of NO and prostaglandins to the acetylcholine (ACh)-induced vasodilation in phenylephrine (PE, 60 μM)-contracted mesenteric resistance arteries. **A** and **B,** Concentration-dependent vasodilation induced by ACh in control conditions and in the presence of 100 μM N^G-nitro-L-arginine (L-NA, panel **A**) or 10 μM indomethacin (panel **B**). **C,** Time course of the relaxation elicited by 100 nM ACh before (control) and after the application of indomethacin. The horizontal bar indicates the period of stimulation. Values are means ± SEM. *, $P < 0.05$ vs control by two-way ANOVA.

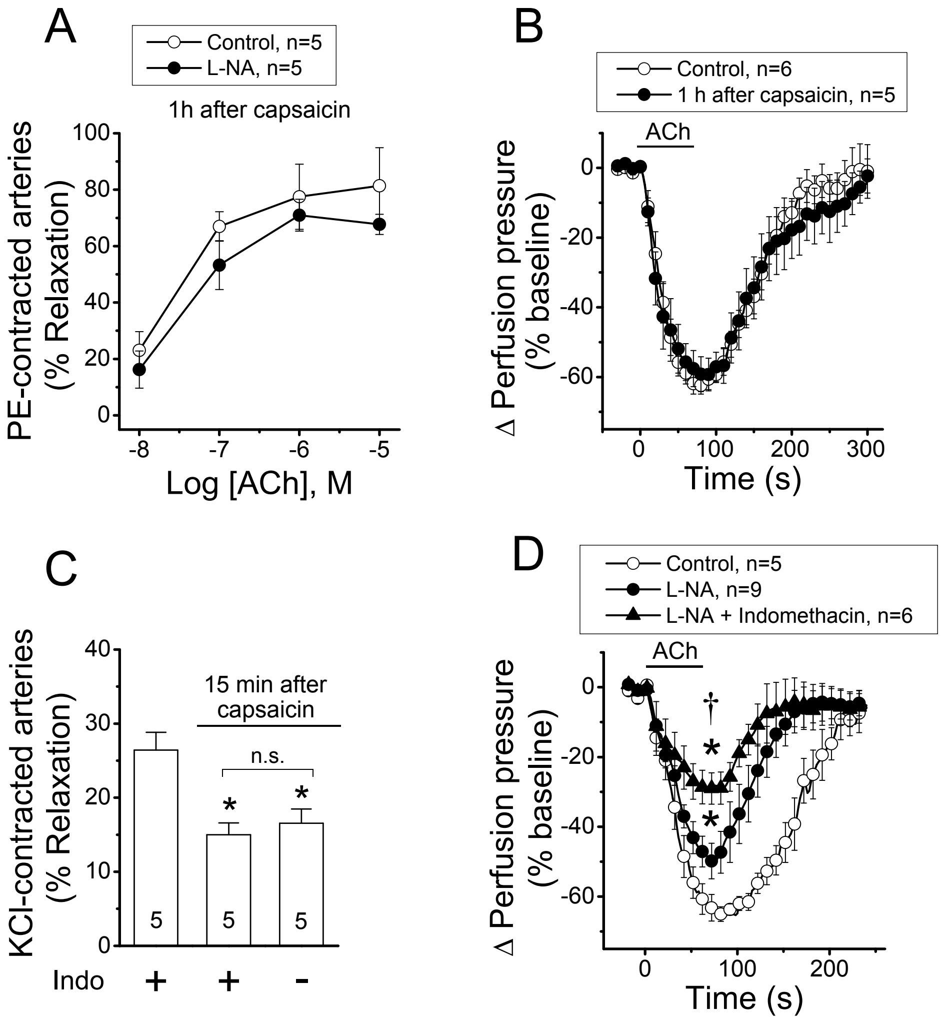

Supplementary Figure S2. Interaction between prostaglandin- and NO-dependent vasodilator components in the capsaicin-initiated inhibition of acetylcholine (ACh)-induced vasodilation. **A,** Concentration-dependent vasodilation induced by ACh in phenylephrine (PE, 60 μM)-contracted mesenteric resistance arteries 1 h after the treatment with 1 μM capsaicin for 20 min. The vasodilator response was assessed in control conditions and after blocking NO production with 100 μM N^G-nitro-L-arginine (L-NA). **B,** Time course of the vasodilation induced by 100 nM ACh in PE (60 μM)-contracted resistance arteries in control conditions and 1 h after the treatment with capsaicin for 20 min. **C,** Effect of the blockade of prostaglandin production with 10 μM indomethacin (Indo) on the maximal vasodilator response activated by ACh 15 min after capsaicin application in KCl (70 mM)-contracted mesenteries. **D,** ACh-induced vasodilation after long-term inhibition of NO production (> 1 h) with L-NA and the effect of the blockade of prostaglandin formation with indomethacin on the response observed in these conditions. ACh-elicited vasodilation was analysed in PE (60 μM)-contracted mesenteric resistance arteries. Note that long-term inhibition of NO production (> 1 h) unmasks an indomethacin-sensitive vasodilator component of the response to ACh, which is not observed in control conditions (**Supplementary Figure S1**). Horizontal bars in B and D indicate the period of stimulation. Numbers inside the bars indicate the n value. Values are means ± SEM. *, $P < 0.01$ vs control and †, $P < 0.01$ vs L-NA by one-way ANOVA plus Newman-Keuls post hoc test.

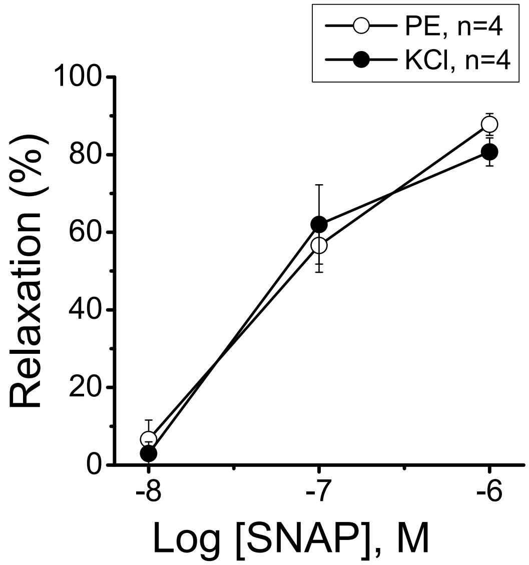

Supplementary Figure S3. NO-dependent vasodilation in phenylephrine (PE)- and KCl-contracted mesenteric resistance arteries. The concentration-dependent vasodilator response induced by SNAP, a NO donor, is shown. Note that the response in phenylephrine (60 μM)-contracted vessels was similar to that observed in KCl (70 mM)-contracted arteries. Values are means ± SEM.

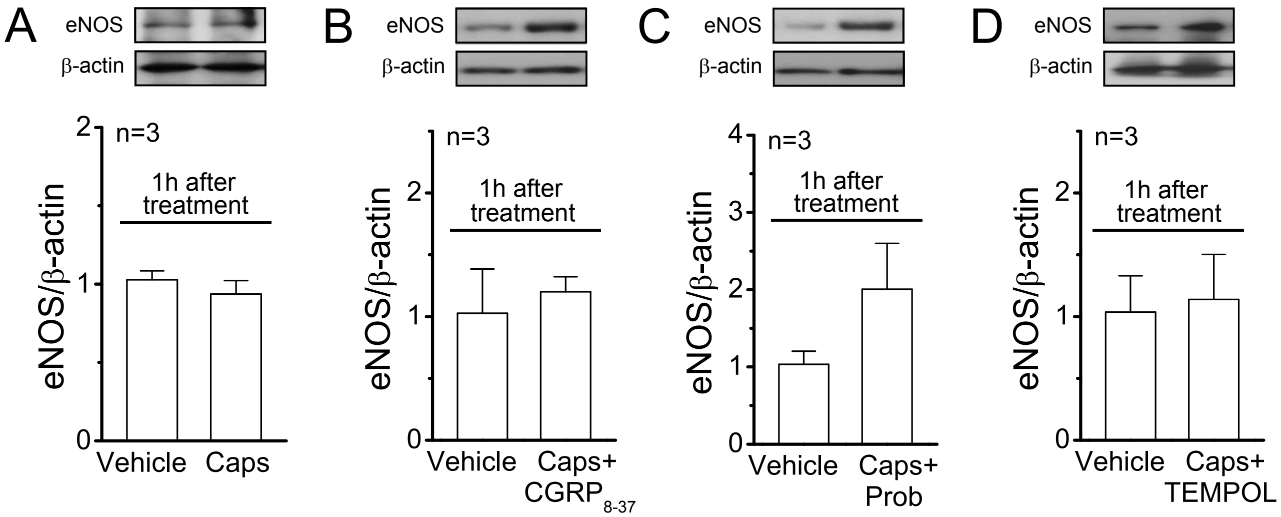

Supplementary Figure S4. Analysis of the effect of capsaicin or the blockade of the capsaicin-initiated signalling pathway on eNOS expression. Representative Western blots and densitometric analysis of the level of eNOS expression observed 1 h after the treatment of mesenteric arteries for 20 min with 1 µM capsaicin (Caps) alone (**A**) or in the presence of $CGRP_{8\text{-}37}$ (**B**), probenecid (Prob, **C**) and TEMPOL (**D**). As control, the effect of the vehicle of Caps is also shown. Changes in protein expression are expressed as the ratio of eNOS over ß-actin. Note that stimulation with Caps in control conditions or during CGRP receptor inhibition with $CGRP_{8\text{-}37}$, pannexin-1-formed channel blockade with Prob or scavenging $O_2^{\bullet -}$ with TEMPOL do not affect eNOS expression. Values are means ± SEM.

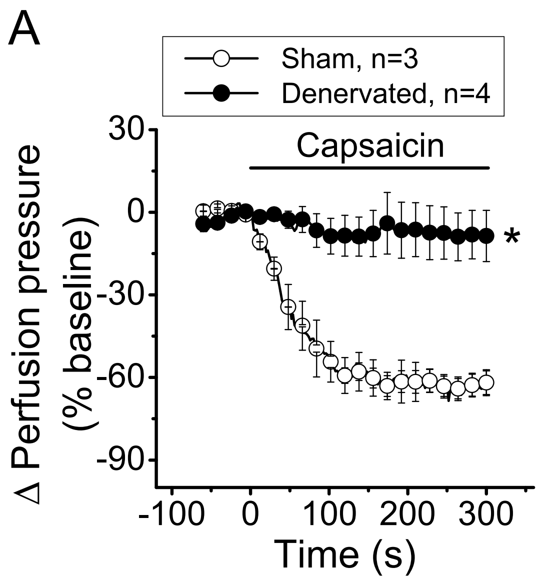

Supplementary Figure S5. Effect of phenol-mediated denervation on the vasodilator response activated by capsaicin and CGRP. **A,** Time course of the capsaicin (1 μM)-induced vasodilation in mesenteric resistance arteries of sham-operated and denervated rats. **B,** Time course of the CGRP (100 nM)-evoked vasodilator response in mesenteric resistance arteries of sham-operated and denervated rats. Note that, as expected, denervation of the mesenteric arterial bed fully prevented the response to capsaicin, but did not reduce the vasodilation elicited by CGRP. Horizontal bars indicate the period of stimulation. Values are means ± SEM. *, $P < 0.05$ vs Sham by two-way ANOVA.

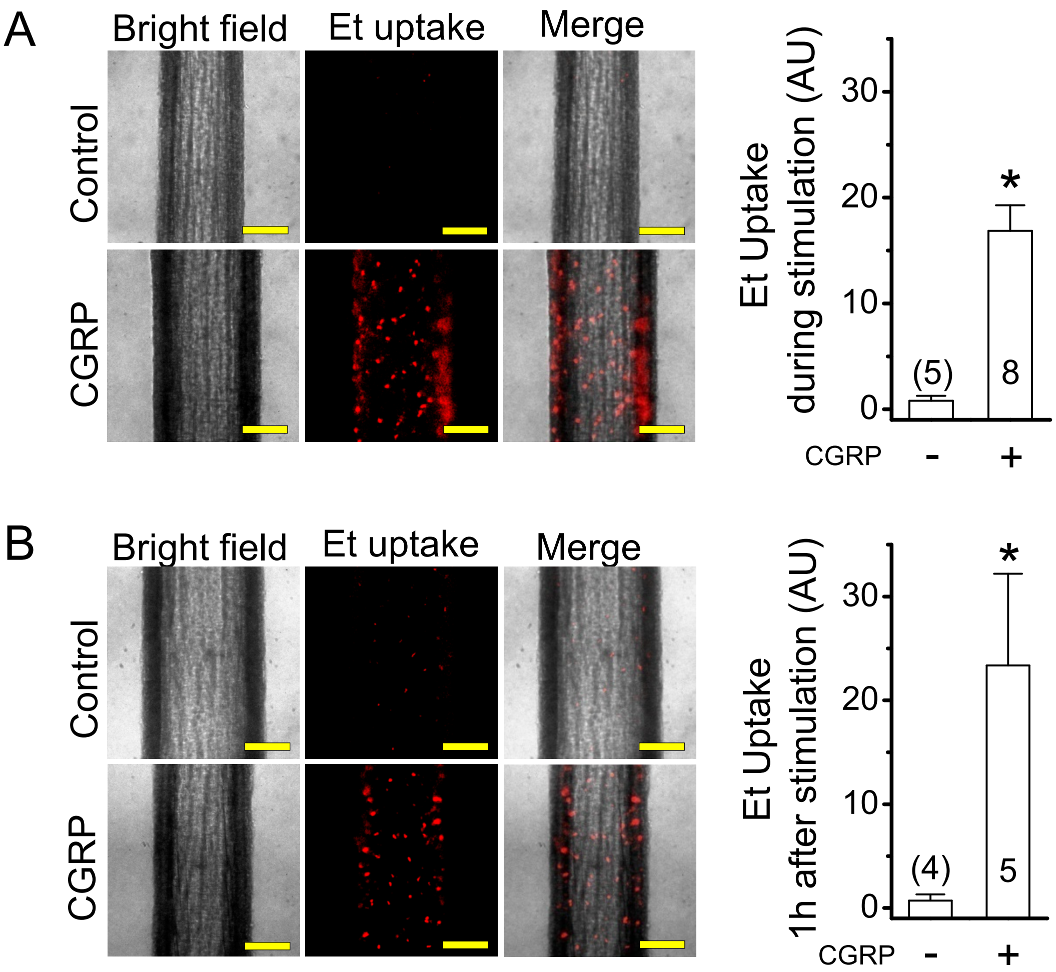

Supplementary Figure S6. Ethidium uptake induced by CGRP in mesenteric resistance arteries. **A,** Representative images and densitometric analysis of the ethidium (Et) uptake observed during the stimulation of mesenteric arteries for 5 min with 100 nM CGRP or its vehicle (Control). **B,** Representative images and densitometric analysis of the ethidium uptake observed 1 h after CGRP application. Dye uptake was assessed by perfusing ethidium for 5 min. Note that, as observed with capsaicin, CGRP-elicited ethidium uptake is prolonged along the time and the magnitude of dye uptake did not decay 1 h after the end of the stimulation period. Numbers inside the bars or in parentheses indicate the n value. Values are means ± SEM. *, $P < 0.05$ vs vehicle by Student's unpaired t-test. Scale bars represent 100 μm.

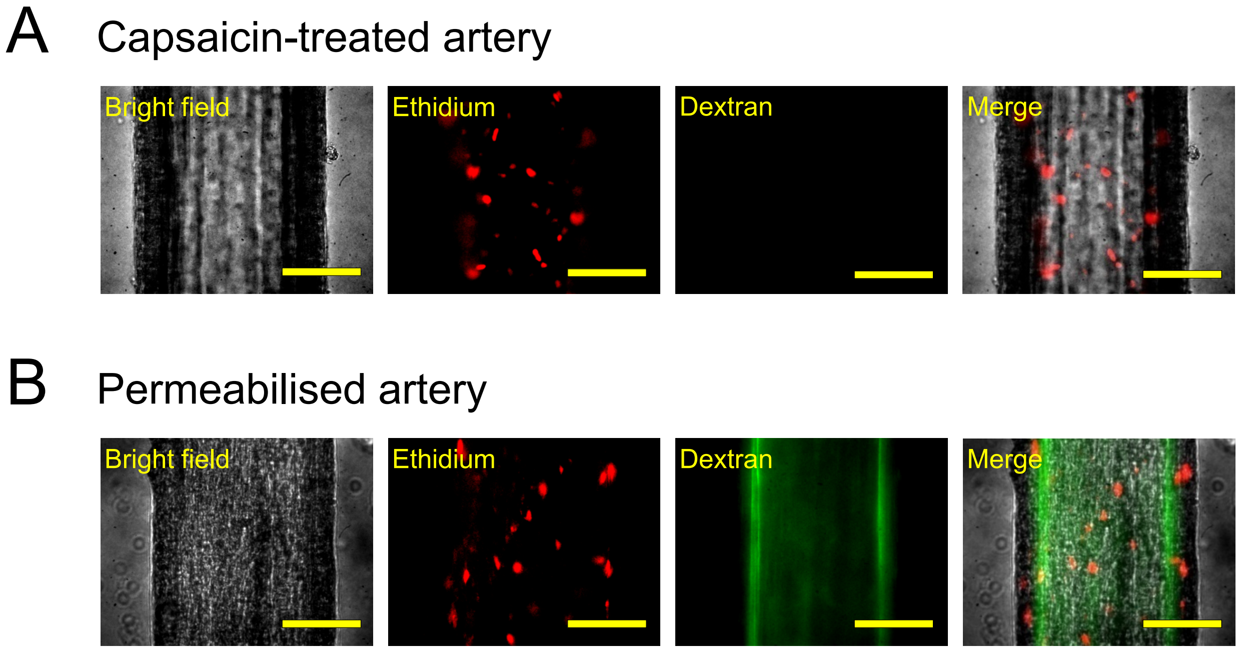

Supplementary Figure S7. The prolonged opening of connexin hemichannels and pannexin-1 channels does not affect the plasma membrane permeability to macromolecules. **A,** Representative images of the ethidium and dextran-FITC (3,000 Da) uptake observed in control mesenteric resistance arteries 1 h after the end of the treatment with 1 μM capsaicin for 20 min. **B,** Representative images of ethidium and dextran-FITC (3,000 Da) uptake attained in Triton X-100 (0.01%) permeabilized mesenteric arteries. Dye uptake was evaluated after perfusing both ethidium and dextran-FITC for 20 min. Scale bars represent 100 μm.

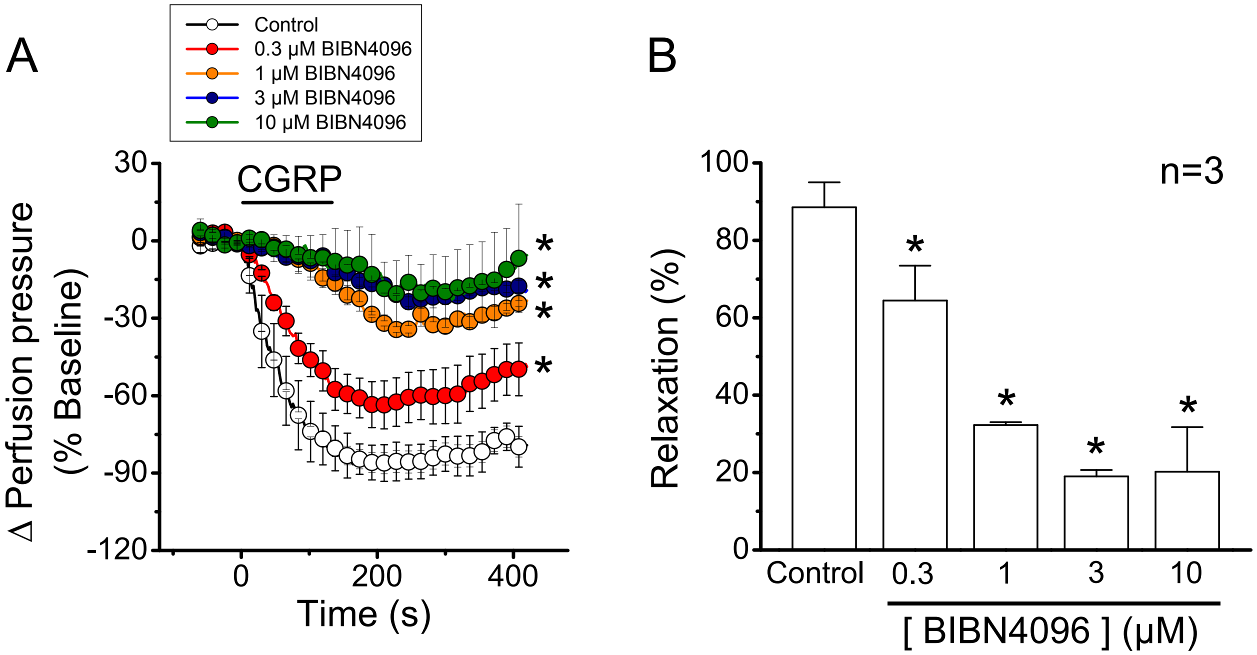

Supplementary Figure S8. Characterization of the inhibitory effect of BIBN4096 on the relaxation induced by CGRP. **A,** Time course of the vasodilation activated by CGRP in the mesenteric arterial bed pre-contracted with 60 µM phenylephrine in control conditions and in the presence of 0.3, 1, 3 and 10 µM BIBN4096. **B,** Analysis of the concentration-dependent inhibition evoked by BIBN4096 on the CGRP-induced maximal relaxation shown in A. The horizontal bar in panel A indicates the period of stimulation. Values are means ± SEM. *, $P < 0.05$ vs control by one-way ANOVA plus Dunnett post hoc test.

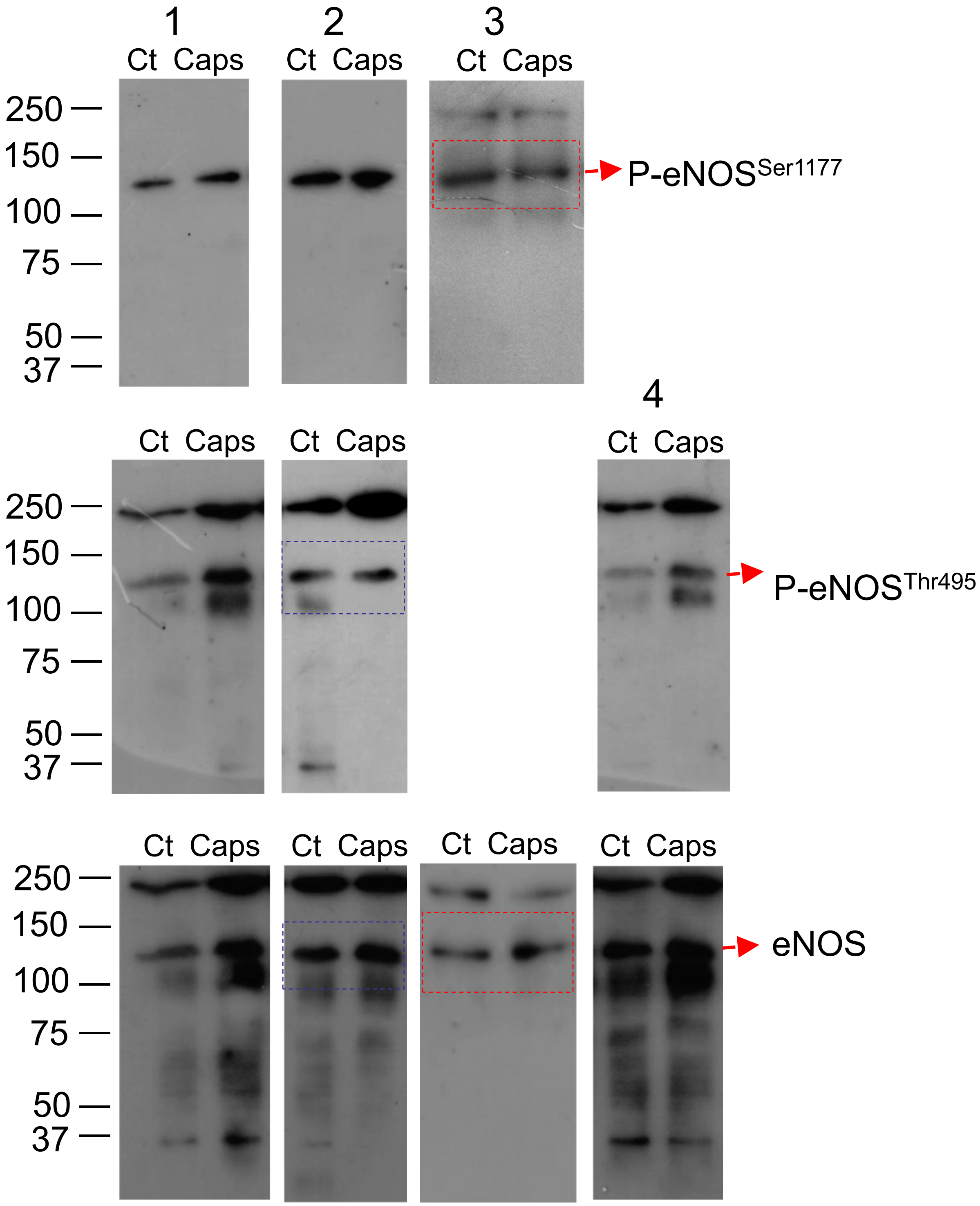

Supplementary Figure S9. Uncropped images of Western blots shown in main Figure 3. Red box areas indicate the cropped regions used as representatives in Figure 3A. Blue box areas indicate the cropped regions used as representatives in Figure 3B. The effect of capsaicin was evaluated in four separate Western blots. The first lane of each Western blot was loaded with a sample of one independent control mesentery homogenized 1 h after stimulation (20 min) with the vehicle of capsaicin (Ct) and the second lane was loaded with a sample of one independent mesentery homogenized 1 h after the treatment (20 min) with 1 μM capsaicin (Caps). Membranes 1 and 2 were first probed for P-eNOS^{Thr495}, and then, stripped twice to detect P-eNOS^{Ser1177} and total eNOS. Membranes 3 and 4 were initially probed for either P-eNOS^{Ser1177} (membrane 3) or P-eNOS^{Thr495} (membrane 4) and for total eNOS after stripping them.

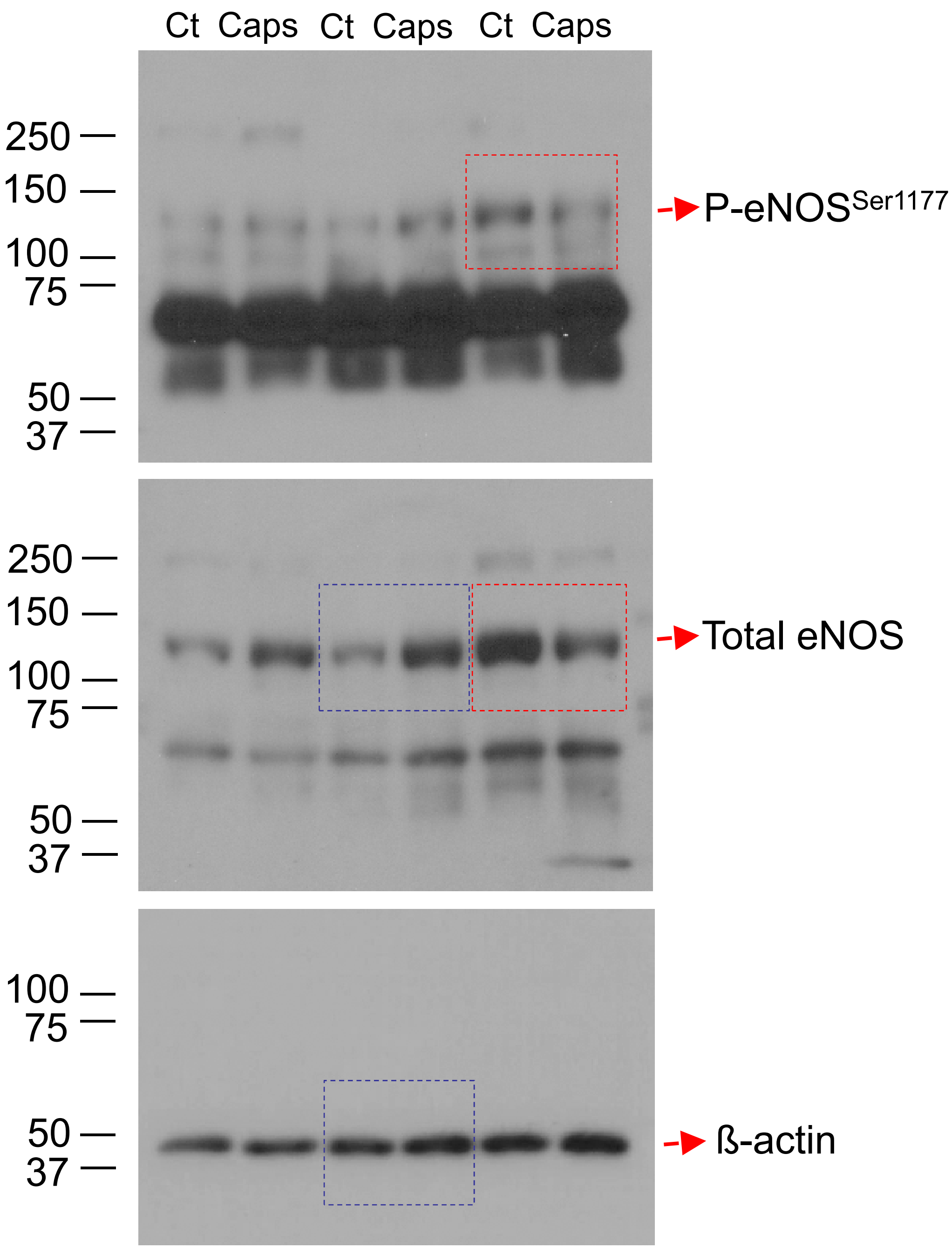

Supplementary Figure S10. Uncropped images of Western blots shown in the main Figure 4C and in the Supplementary Figure S4B. Red box areas indicate the cropped regions used as representatives in Figure 4C. Blue box areas indicate the cropped regions used as representatives in Supplementary Figure S4B. Lanes 1, 3 and 5 were loaded with samples of 3 independent control mesenteries homogenized 1 h after stimulation (20 min) with the vehicle of capsaicin (Ct). Lanes 2, 4 and 6 were loaded with samples of 3 independent mesenteries homogenized 1 h after the treatment (20 min) with 1 μM capsaicin (Caps) in the presence of $CGRP_{8\text{-}37}$. The same membrane was initially probed for P-$eNOS^{Ser1177}$ (top panel), and then, stripped twice to detect total eNOS (middle panel) and ß-actin (bottom panel).

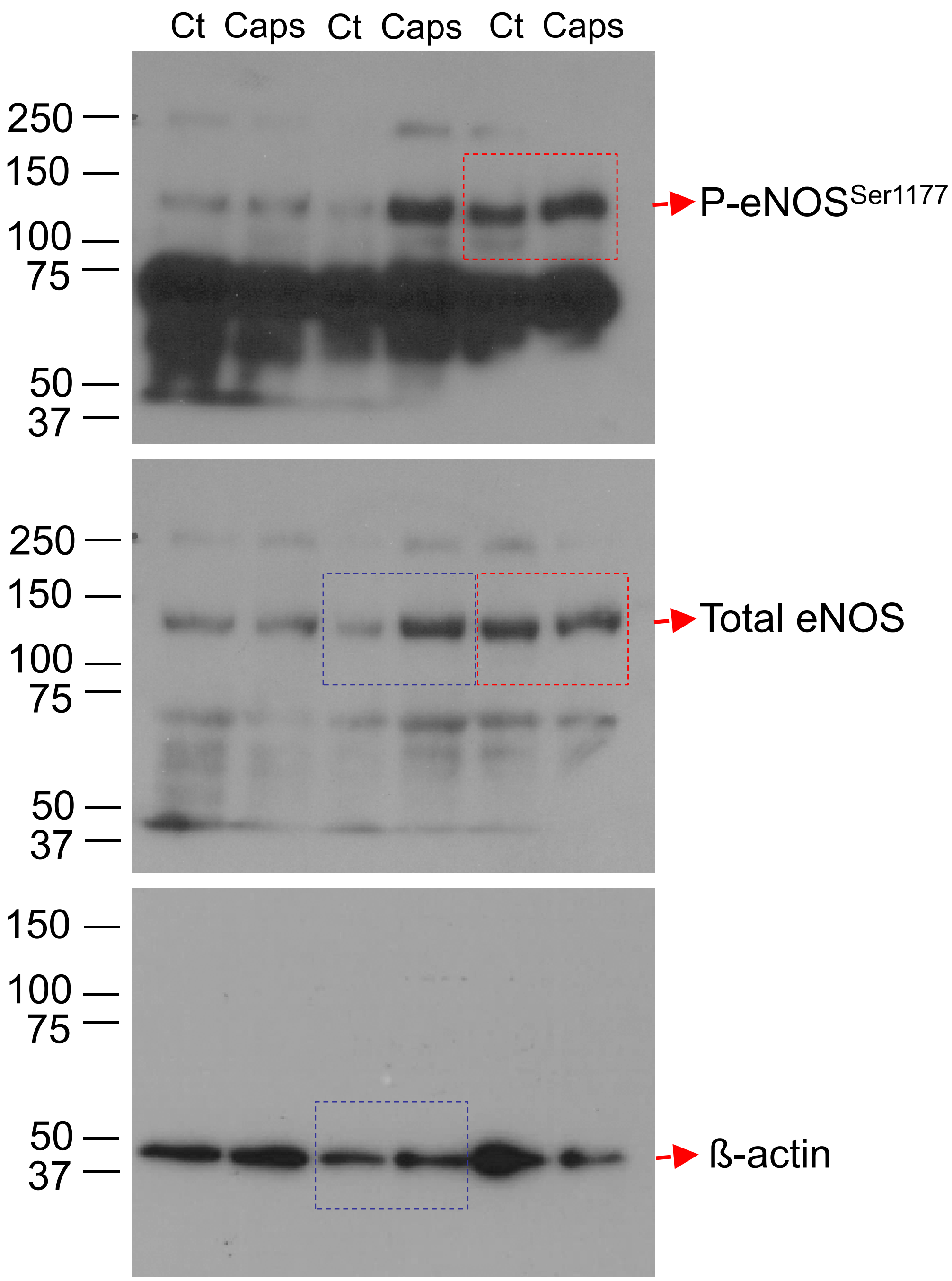

Supplementary Figure S11. Uncropped images of Western blots shown in the main Figure 8A and in the Supplementary Figure S4C. Red box areas indicate the cropped regions used as representatives in Figure 8A. Blue box areas indicate the cropped regions used as representatives in Supplementary Figure S4C. Lanes 1, 3 and 5 were loaded with samples of 3 independent control mesenteries homogenized 1 h after stimulation (20 min) with the vehicle of capsaicin (Ct). Lanes 2, 4 and 6 were loaded with samples of 3 independent mesenteries homogenized 1 h after the treatment with 1 µM capsaicin (Caps) in the presence of probenecid. The same membrane was initially probed for P-eNOS^{Ser1177} (top panel) and then stripped twice to detect total eNOS (middle panel) and ß-actin (bottom panel).

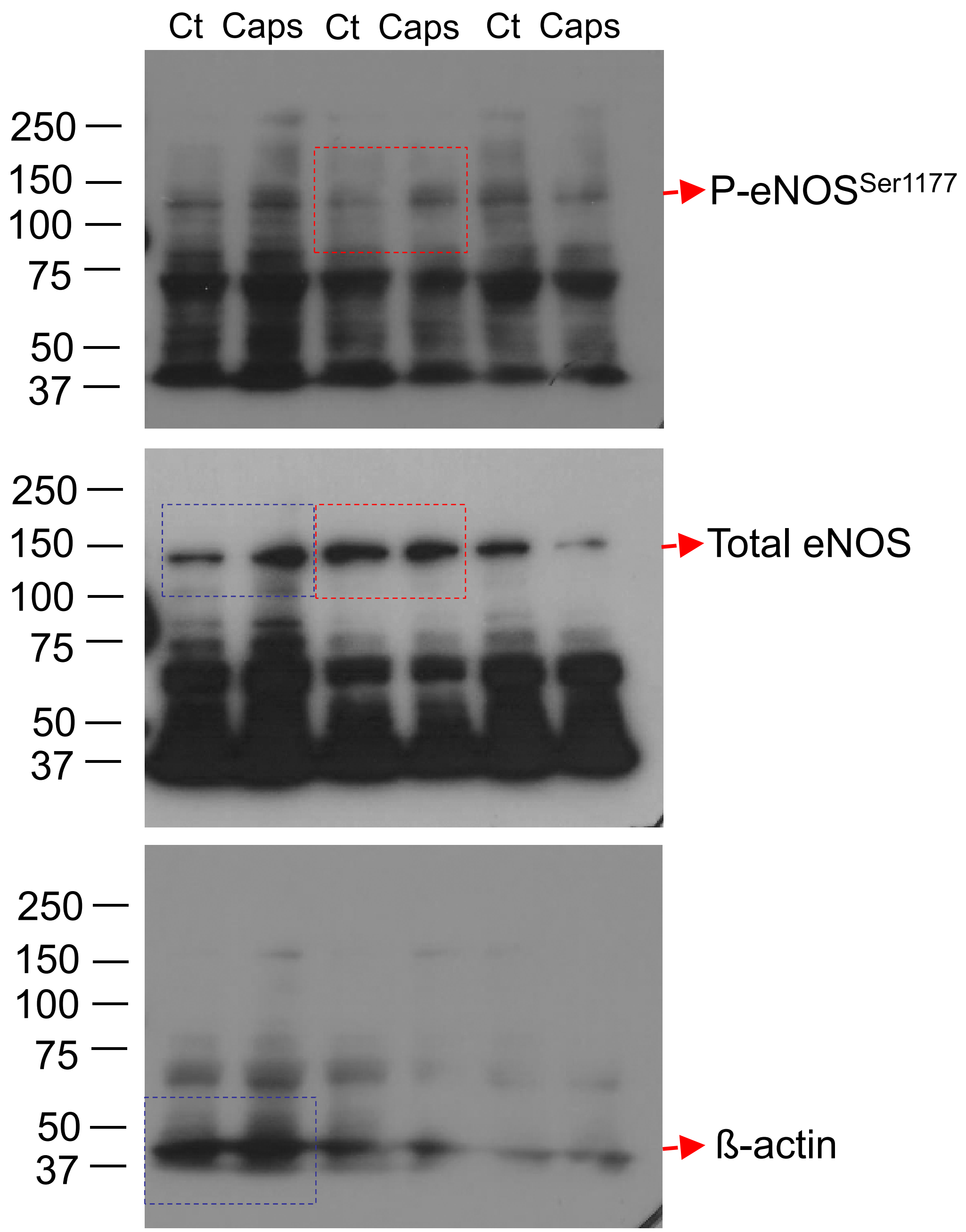

Supplementary Figure S12. Uncropped images of Western blots shown in the main Figure 8B and in the Supplementary Figure S4D. Red box areas indicate the cropped regions used as representatives in Figure 8B. Blue box areas indicate the cropped regions used as representatives in Supplementary Figure S4D. Lanes 1, 3 and 5 were loaded with samples of 3 independent control mesenteries homogenized 1 h after stimulation (20 min) with the vehicle of capsaicin (Ct). Lanes 2, 4 and 6 were loaded with samples of 3 independent mesenteries homogenized 1 h after the treatment (20 min) with 1 µM capsaicin (Caps) in the presence of TEMPOL. The same membrane was initially probed for P-eNOS^{Ser1177} (top panel) and then stripped twice to detect total eNOS (middle panel) and ß-actin (bottom panel).

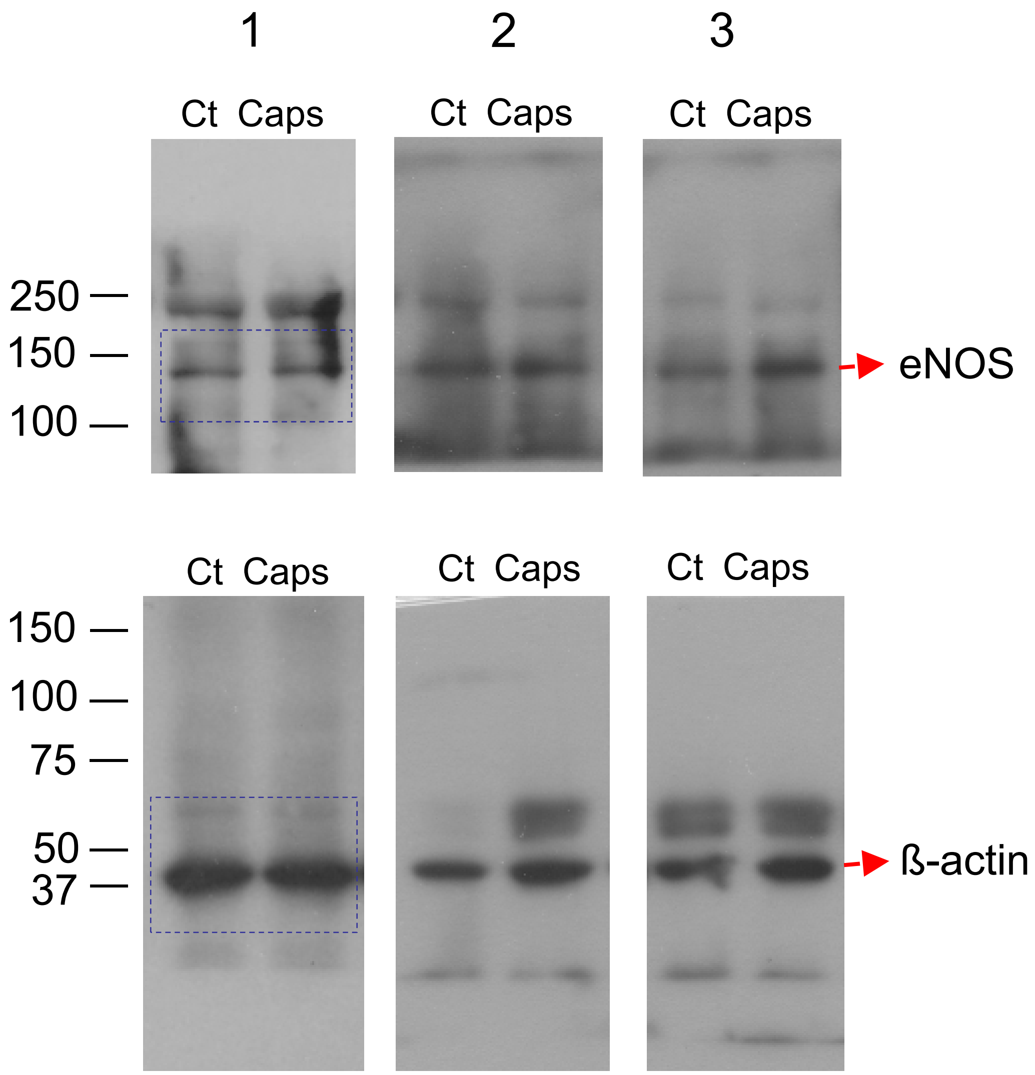

Supplementary Figure S13. Uncropped images of Western blots shown in Supplementary Figure S4A. Blue box areas indicate the cropped regions used as representatives in Supplementary Figure S4A. The effect of capsaicin was evaluated in three separate Western blots. The first lane of each Western blot was loaded with a sample of one independent control mesentery homogenized 1 h after stimulation (20 min) with the vehicle of capsaicin (Ct) and the second lane was loaded with a sample of one independent mesentery homogenized 1 h after the treatment (20 min) with 1 µM capsaicin (Caps). Membranes were first probed for eNOS (top panel), and then, stripped to detect ß-actin (bottom panel).
